# A Novel Radiotherapeutic Approach to Treat Bulky Metastases Even From Cutaneous Squamous Cell Carcinoma: Its Rationale and a Look at the Reliability of the Linear-Quadratic Model to Explain Its Radiobiological Effects

**DOI:** 10.3389/fonc.2022.809279

**Published:** 2022-02-23

**Authors:** Gianluca Ferini, Paolo Castorina, Vito Valenti, Salvatore Ivan Illari, Ilias Sachpazidis, Luigi Castorina, Maurizio Marrale, Stefano Pergolizzi

**Affiliations:** ^1^ Department of Radiation Oncology, REM Radioterapia srl, Viagrande, Italy; ^2^ Istituto Oncologico del Mediterraneo, Viagrande, Italy; ^3^ Faculty of Mathematics and Physics, Charles University, Prague, Czechia; ^4^ Istituto Nazionale Fisica Nucleare, Catania, Italy; ^5^ Department of Radiation Oncology, Fondazione IOM, Viagrande, Italy; ^6^ Department of Radiation Oncology, Division of Medical Physics, Medical Centre, Faculty of Medicine, University of Freiburg, Freiburg, Germany; ^7^ Department of Research & Development, Medical Innovation and Technology P. C., Mesolongi, Greece; ^8^ Department of Physics and Chemistry, “Emilio Segrè” ATeN Center, University of Palermo, Palermo, Italy; ^9^ Istituto Nazionale di Fisica Nucleare (INFN), Sezione di Catania, Catania, Italy; ^10^ Dipartimento di Scienze Biomediche, Odontoiatriche e delle Immagini Morfologiche e Funzionali Università di Messina, Messina, Italy

**Keywords:** lattice radiotherapy, tumor control probability (TCP), normal tissue complication probability (NTCP), spatially fractionated radiation therapy, immunotherapy, metabolic tumor volume, bulky tumors, cutaneous squamous cell carcinoma

## Abstract

**Introduction:**

Metastatic cutaneous squamous cell carcinoma (cSCC) is a very rare condition. The lack of definition of an oligometastatic subgroup means that there is no consensus for its treatment, unlike the mucosal head and neck counterpart. Like the latter, the cutaneous form is able to develop bulky tumor masses. When this happens, the classic care approach is just for palliative intent due to a likely unfavorable benefit–risk balance typical of aggressive treatments. Here we proposed a novel radiotherapy (RT) technique to treat bulky metastases from cSCC in the context of an overall limited tumor burden and tried to explain its clinical outcome by the currently available mathematical radiobiological and *ad hoc* developed models.

**Methods:**

We treated a case of facial cSCC with three metastases: two of them by classic stereotactic RT and the other by lattice RT supported by metabolic imaging (^18^F-FDG PET) due to its excessively large dimensions. For the latter lesion, we compared four treatment plans with different RT techniques in order to define the best approach in terms of normal tissue complication probability (NTCP) and tumor control probability (TCP). Moreover, we developed an *ad hoc* mathematical radiobiological model that could fit better with the characteristics of heterogeneity of this bulky metastasis for which, indeed, a segmentation of normoxic, hypoxic, and necrotic subvolumes might have been assumed.

**Results:**

We observed a clinical complete response in all three disease sites; the bulky metastasis actually regressed more rapidly than the other two treated by stereotactic RT. For the large lesion, NTCP predictions were good for all four different plans but even significantly better for the lattice RT plan. Neither the classic TCP nor the *ad hoc* developed radiobiological models could be totally adequate to explain the reported outcome. This finding might support a key role of the host immune system.

**Conclusions:**

PET-guided lattice RT might be safe and effective for the treatment of bulky lesions from cSCC. There might be some need for complex mathematical radiobiological models that are able to take into account any immune system’s role in order to explain the possible mechanisms of the tumor response to radiation and the relevant key points to enhance it.

## Introduction

The main aim of reporting this experience is to inform insiders about the possibility to safely and aggressively irradiate difficult-to-treat bulky tumors, even large metastases from cutaneous squamous cell carcinoma (cSCC), with a particular radiotherapeutic option. An interpretation of its clinical results is provided, as well as of its ambiguities, likely needing new radiobiological investigations.

Facial cSCC is one of the most frequent skin cancers, especially among elderly patients who most commonly report a history of prolonged occupational exposure to ultraviolet radiation from sunlight (1). It is able to develop disfiguring and ulcerating bulky lesions and regional lymph nodes and, occasionally, distant metastases through the bloodstream (2). Oligometastatic status was described for the mucosal counterpart of the head and neck squamous cell carcinoma (HNSCC), but not for its cutaneous form (3). However, these two variants are often grouped together in some cancer registries (4, 5) and share some chemo- and immunotherapy regimens in locally advanced/metastatic stages (6). The rarity of the metastatic disease and the low risk of cancer-specific death for cSCC (7) do not allow to rule out the existence of an oligometastatic stage whereby radiotherapy (RT) could be employed with a curative purpose, as it has been successfully done for other metastatic cancers by adopting the stereotactic approach (8–10). Moving from a low palliative radiation dose prescription toward a higher radical one causes some concerns about normal tissue tolerance, especially for the treatment of bulky tumors. Spatially fractionated RT (SFRT), specifically lattice RT, overcomes such an issue by delivering a highly heterogeneous radiation dose to large targets in order to spare the neighboring organs at risk (OARs) (11). Such a peculiar dose delivery method could face the typical non-homogeneous tumor growth by selecting the hypoxic regions to be boosted for overcoming their relative radioresistance (12). In these scenarios, choosing the best dose prescription could be very difficult for the radiation oncologist who must strike a balance between an optimal tumor control probability (TCP) and an acceptable NTCP (13). Actually, the classic RT protocols can be unable to achieve a clinical complete response (cCR), likely due to a radiation dose insufficient to eradicate any residual cancer cell (14). Tumor behavior may be described by mathematical models, as regards both the initial cancer cell proliferation and the repopulation dynamics following the oncologic treatments. Some of these models, including the popular linear-quadratic (LQ) one, can assist the radiation oncologist in the radiobiological determination of dose escalation to improve TCP, as well as of the most suitable fraction size and interval to avoid normal tissue damage (15).

**Figure 1:**
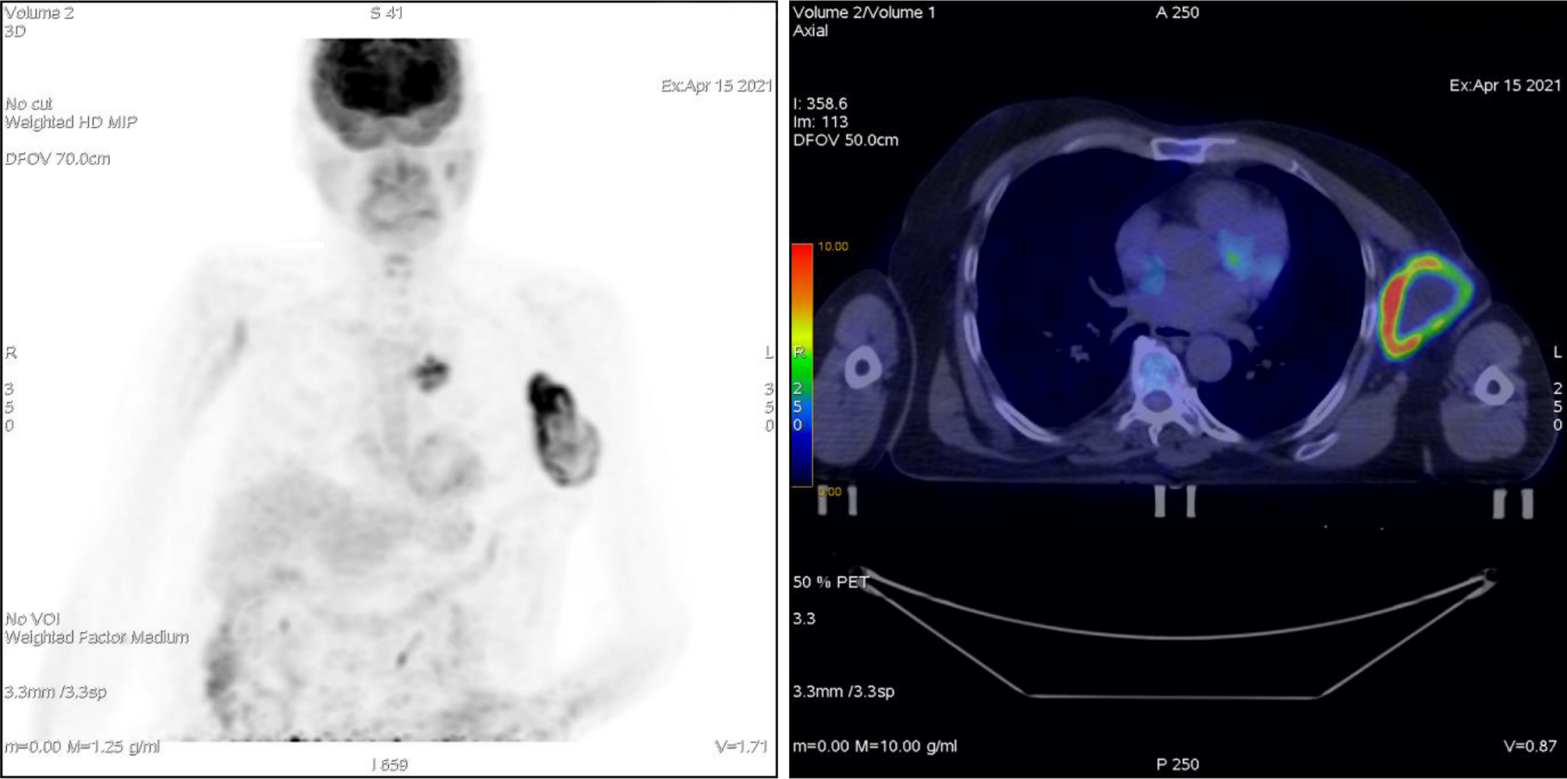
Patient ^18^F-FDG PET at presentation.

Here we report a case of metastatic facial cSCC with three separate distant lesions, two of which were radically treated with stereotactic body RT (SBRT) and the remaining one with lattice RT (**Figure 1**). For the latter case, we present four different treatment plans that are inter-compared by dose volume histograms (DVHs). Furthermore, in order to evaluate the effectiveness of each plan, TCP values according to Poisson’s model as well as NTCP distributions according to the Lyman–Kutcher–Burman (LKB) model were calculated. Furthermore, we developed a numerical analysis based on the cell regrowth model to try to explain some aspects of the observed clinical outcome taking also into account the tumor heterogeneity.

## Case Presentation

**Figure 2:**
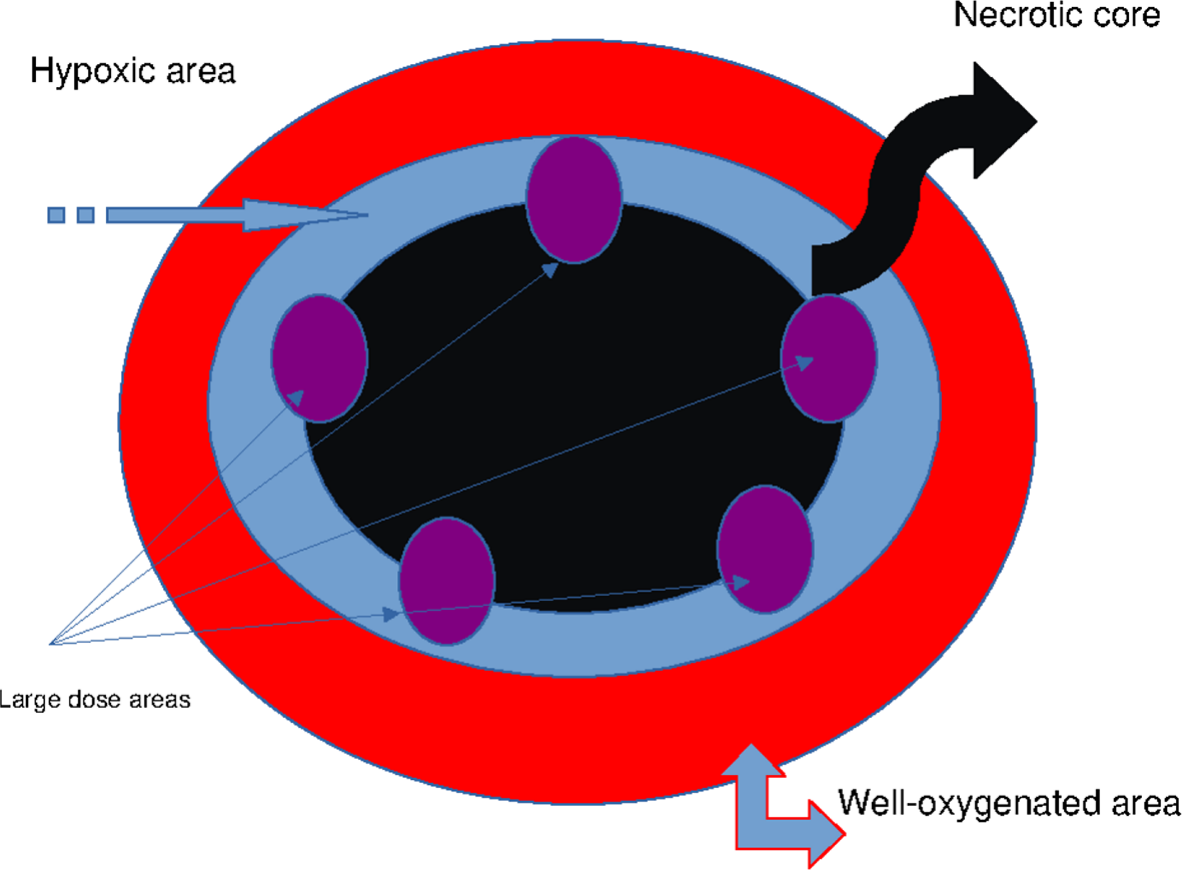
Schematic representation of the axillary GTV: necrotic core (black), mitotic area (red), whole hypoxic area (blue), and vertices targeted by large dose boost (purple). GTV, gross tumor volume.

**Figure 3:**
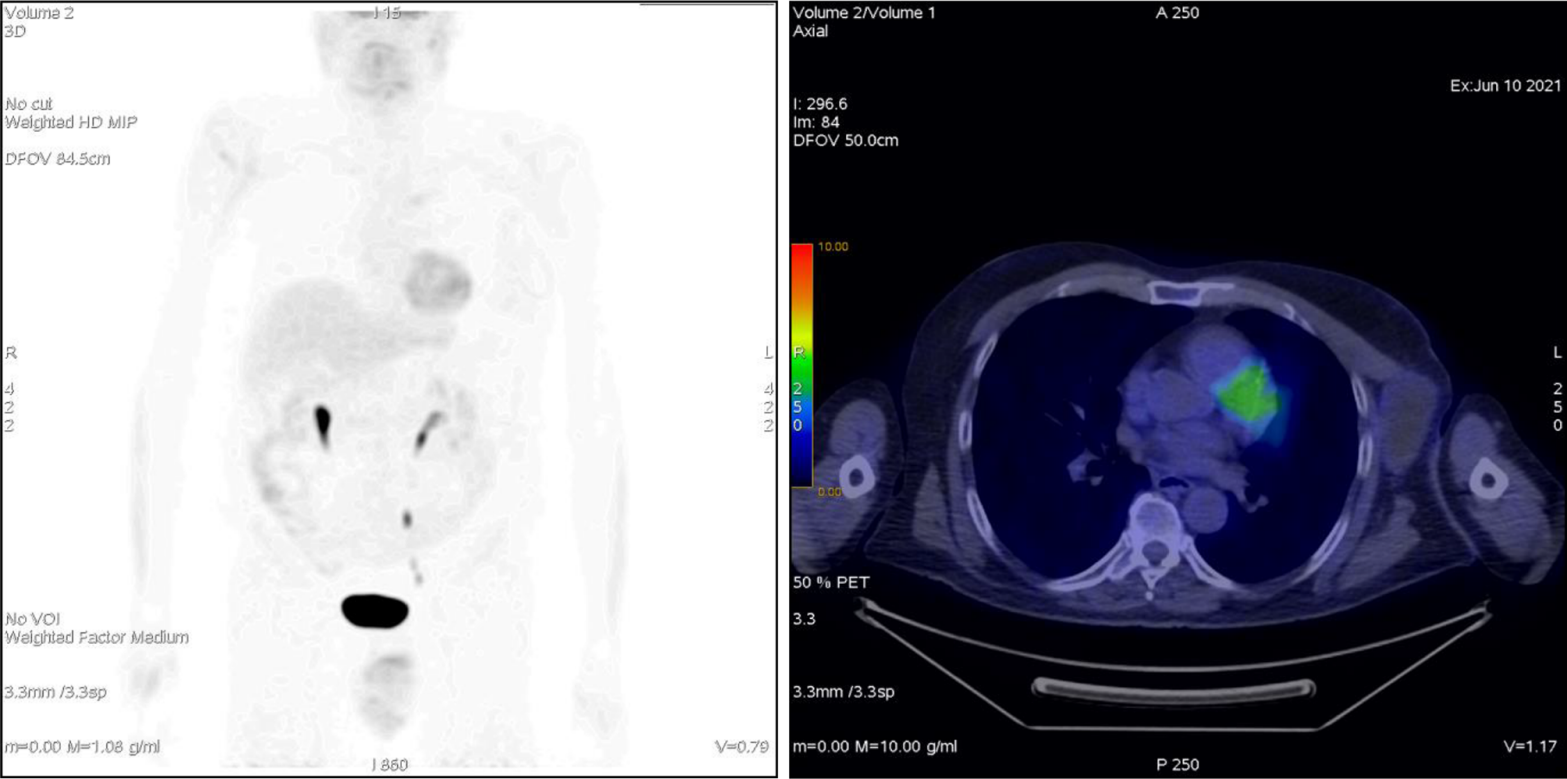
Patient ^18^F-FDG PET 1 month after treatment.

**Figure 4:**
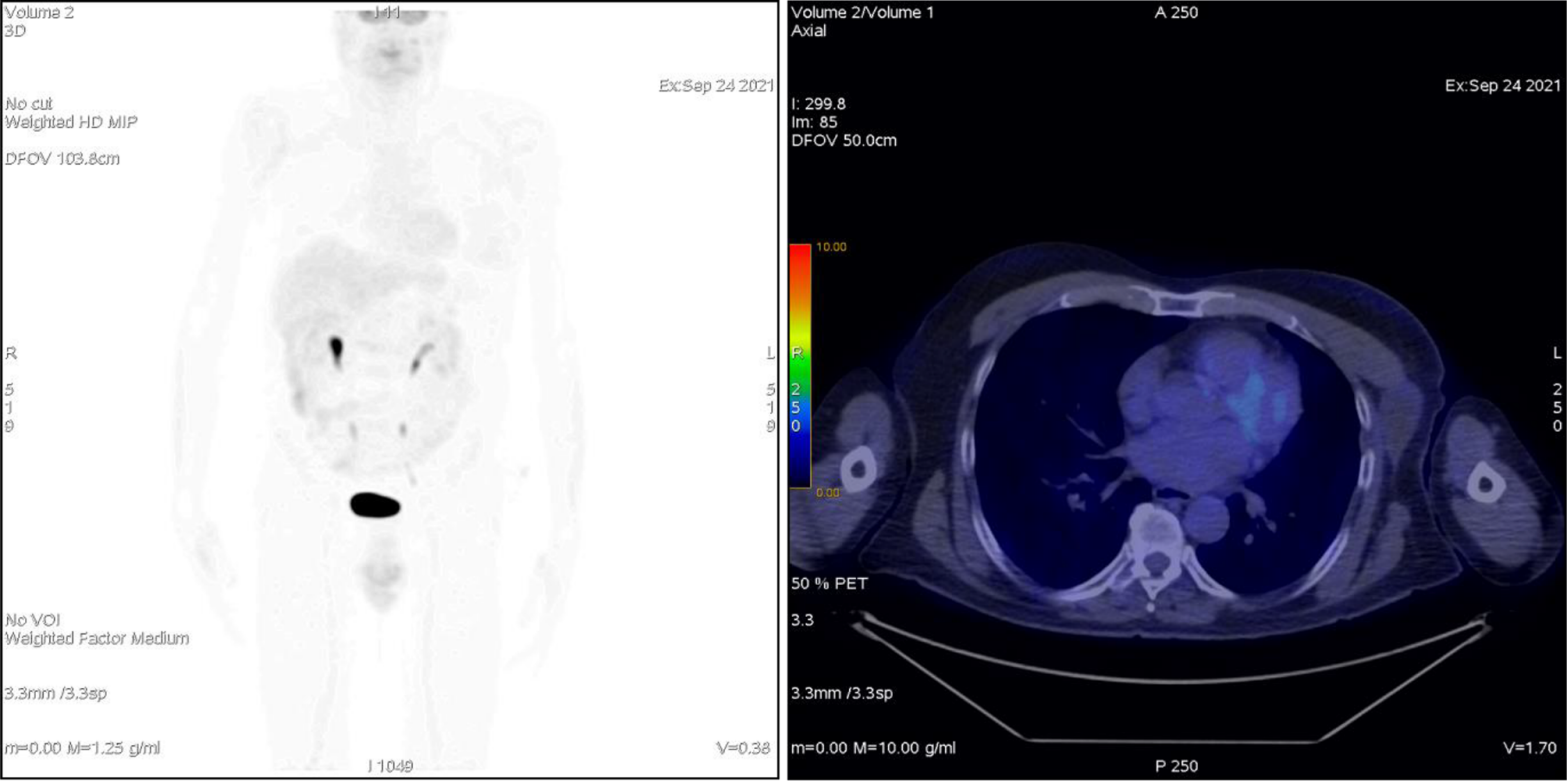
Patient ^18^F-FDG PET at 4.5 months after treatment.

**Figure 5:**
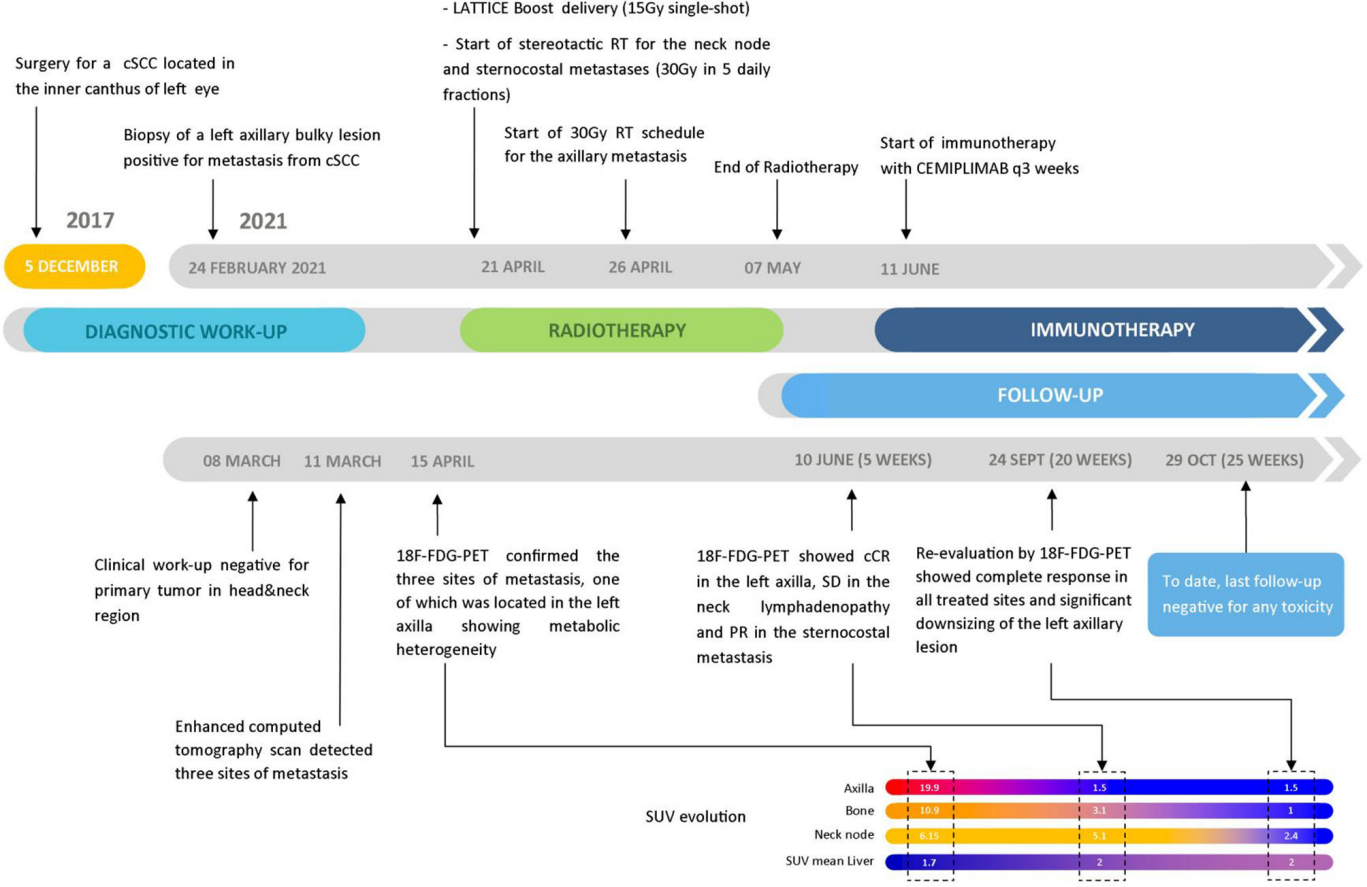
Patient’s timeline from the beginning of the disease to the last follow-up accompanied by the SUV evolution of the ^18^F-FDG PET scans through the various steps.

A 75-year-old patient with no significant comorbidities was submitted to surgical removal of a growing reddish and hard skin nodule located on the inner canthus of the left eye in December 2017. The histology report showed a moderately differentiated cSCC, which was totally resected with negative margins (R0). No adjuvant therapies were deemed necessary since it was an early-stage cancer. Long-term follow-up was negative up to February 2021, when the patient required medical attention due to the appearance of a suspicious red, painful, and semi-soft lump in the left axilla. The nature of such a lesion was then clarified by means of a needle biopsy, which found the same histology of the previous facial tumor. A complete head and neck clinical workup (i.e., physical and fiber-optic examination) allowed us to exclude a mucosal origin. A contrast-enhanced CT scan showed that the palpable axillary lump was just the tip of an inhomogeneous bulky lesion with a semi-fluid core [maximum diameter was 10.4 cm, volume 171.3 cm^3^, Hounsfield units range −115 to 24.4 ( ± 17.3)] and revealed two further lesions: one was lymphadenopathy of 2-cm diameter located at the II left Robbins level of the neck and the other was a painless metastasis of 3.5-cm diameter at the second left sternocostal joint. An ^18^F-FDG PET confirmed the three sites of metastatic disease. The axillary bulky lesion had a highly inhomogeneous radioactive tracer distribution due to the presence of a “photopenic area” (SUVmean 0.9) in the inner region, corresponding to the low-density area [Hounsfield units range −100 to 22.9 ( ± 13.8)] on the CT image: such characteristics suggested a necrotic core, surrounded by a super-avid actively proliferating thick ring, in a way that we were able to segment two subvolumes for this lesion (**Figure 1**). We attributed such differences to a heterogeneous oxygen landscape within the tumor. Consequently, we named three concentric subvolumes (**Figure 2**): the innermost was the “necrotic core” (86.8 cm^3^), the outermost was the “normoxic subvolume” (71.5 cm^3^), and the transitional mid-layer was arbitrarily established as the “hypoxic subvolume” (13 cm^3^) in an analogous way as previously done by Tubin et al. (16). The latter volume was derived from a 2-mm isometric expansion of the clinically detected necrotic area from which then a ring-shaped subvolume has been subtracted. We considered the disease as in oligometastatic status and proposed an aggressive treatment, keeping us away from a purely palliative intent. The two smallest lesions were treated with SBRT for a total dose of 30 Gy in five consecutive daily fractions of 6 Gy. We deemed the bulky axillary lesion as not approachable by SBRT due to its exceeding size. Therefore, we decided to treat this lesion with SFRT by using a single-shot dose of 15 Gy precisely conformed to five small vertices, followed by 30 Gy in 10 daily fractions of 3 Gy delivered to the entire gross volume. The patient completed the RT schedule in 17 days in total (from April 21 to May 7) with no toxicity. An ^18^F-FDG PET was performed 1 month later for a very early assessment of tumor response to treatment: according to the PERCIST criteria (17), the neck node was stable, sternocostal metastasis had a partial response (PR), and the bulky axillary lesion had a complete response. The co-registered CT scan documented stable disease for the lymphadenopathy, a slight increase in the skeletal lesion (still fitting the RECIST criteria for stable disease), and a smaller residual liquid-like axillary mass without any solid component all around (**Figure 3**). Five weeks after completion of RT, the patient started systemic treatment with cemiplimab (350 mg i.v. q3weeks administered by intravenous infusion over 30 min). After 15 weeks from the start of cemiplimab (20 weeks after the end of RT), an ^18^F-FDG PET/CT was performed for a new assessment of tumor response, showing a complete response in both neck node and sternocostal metastases; the axillary lesion maintained a complete absence of pathological metabolism. On a co-registered CT scan, a further reduction in the size of the axillary mass was detected (from the initial size mm 70 × 46 × 104 to mm 52 × 35 × 83) (**Figure 4**). At the last follow-up, October 29, 2021 (after 25 weeks from the end of irradiation), the patient developed no treatment-related toxicity and complete pain relief in the axillary site. Cemiplimab was well-tolerated. The patient’s timeline is shown in **Figure 5**.

## Methods

### Target Volume Definition

A neck and thorax 1.25-mm thickness slice CT simulation without contrast medium was performed after modeling a thermoplastic mask on the patient for immobilization of the head, neck, and shoulders in a reproducible setup. Regarding the metabolic imaging, PET/CT scans were acquired with a GE Discovery five-ring PET tomography 50 min after i.v. of 185 MBq of ^18^F-FDG and processed with software Q Clear. Thanks to this software, it was possible to calculate not only the SUVmax and the SUVmean but also the metabolic tumor volume (MTV) and the total lesion glycolysis (TLG = MTV × SUVmean).

**Figure 6:**
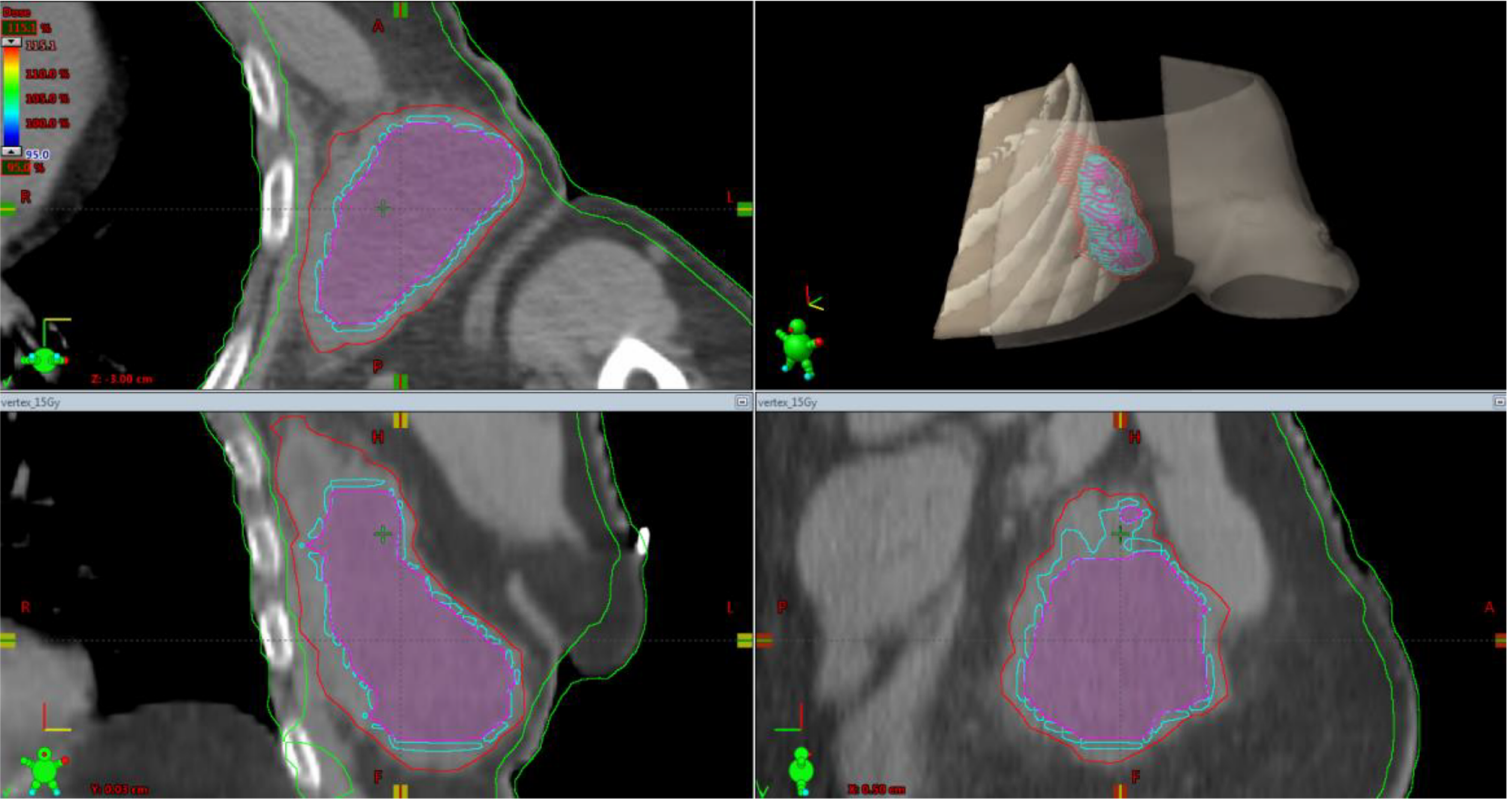
Simulation CT scan images in the axial (top left), coronal (lower left), sagittal (lower right) planes. In the top right picture, a 3D rendering of the spatial relations of target subvolumes with the skin and chest wall (portions included in the NTCP calculation) is shown. The purple line encloses the necrotic subvolume, the light-blue one represents the hypoxic ring, the red line contains the gross tumor volume, and the green lines are for OARs, i.e., chest wall and skin. NTCP, normal tissue complication probability; OARs, organs at risk.

For the left axillary site, the gross tumor volume (GTV) was the entire bulky lesion as defined on the CT scan. We contoured three concentric subvolumes: the innermost was the “necrotic core” (86.8 cm^3^), the outermost the “normoxic subvolume” (71.5 cm^3^), and the transitional mid-layer was arbitrarily established as the “hypoxic subvolume” (13 cm^3^) (**Figure 6**). The vertices were five spheres of 1-cm diameter, astride the boundary between the metabolically active external ring and the necrotic core, that is, where a transitional hypoxic zone may be assumed. The other two GTVs (neck lymph node and sternocostal joint) were defined on the CT images with the support of an ^18^F-FDG PET. Three clinical target volumes (CTVs) were created by an isometric expansion of 0.5 cm for each GTV in order to target also the subclinical disease around the macroscopic one.

### Treatment Planning

For the axillary bulky lesion, three couple of plans were generated using Treatment Planning Software Eclipse^®^ (version 13.7.14 powered by Varian) in a Volumetric Modulated Arc Therapy (VMAT) technique; the treatment unit used for this work was a Novalis-TrueBeam STx linear accelerator equipped with a high-definition multi-leaf collimator (MLC) and an X-ray image guidance system including a six degrees of freedom robotic couch (ExacTrac, BrainLab^®^, Munich, Germany). The high-dose vertex volume was arbitrarily configured using five spherical high-dose vertices with a diameter of 1.0 cm placed within the GTV and with at least 2.0 cm of separation (center to center). The optimized monoisocentric plan resulted in at least 99% of the prescribed dose covering 100% of each vertex volume (D100vertex ≥ 14.85 Gy).

Each couple of plans was made a planned sum.

**Figure 7:**
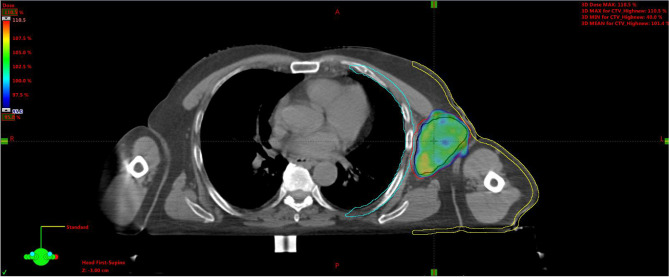
Plan A: 30 Gy to the entire CTV. Black line is for GTV, red for CTV, light-blue for the chest wall, and yellow for the skin. CTV, clinical target volume; GTV, gross tumor volume.

**Figure 8:**
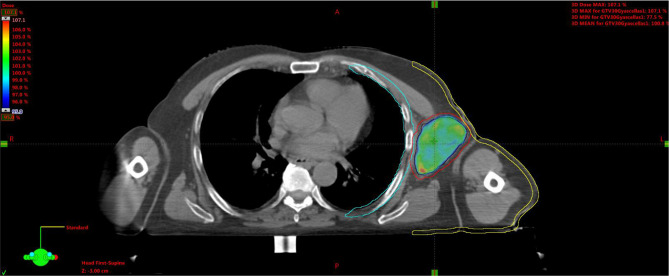
Plan B: 15 Gy to the GTV. GTV, gross tumor volume.

**Figure 9:**
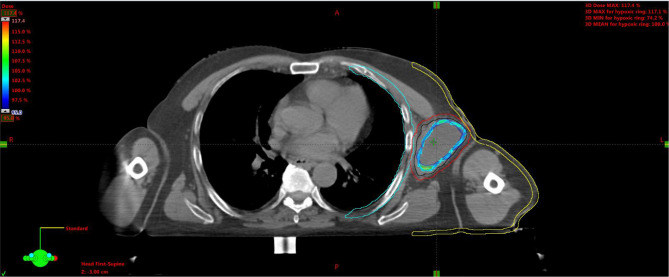
Plan C: 15 Gy to the hypoxic ring (light-blue area).

**Figure 10:**
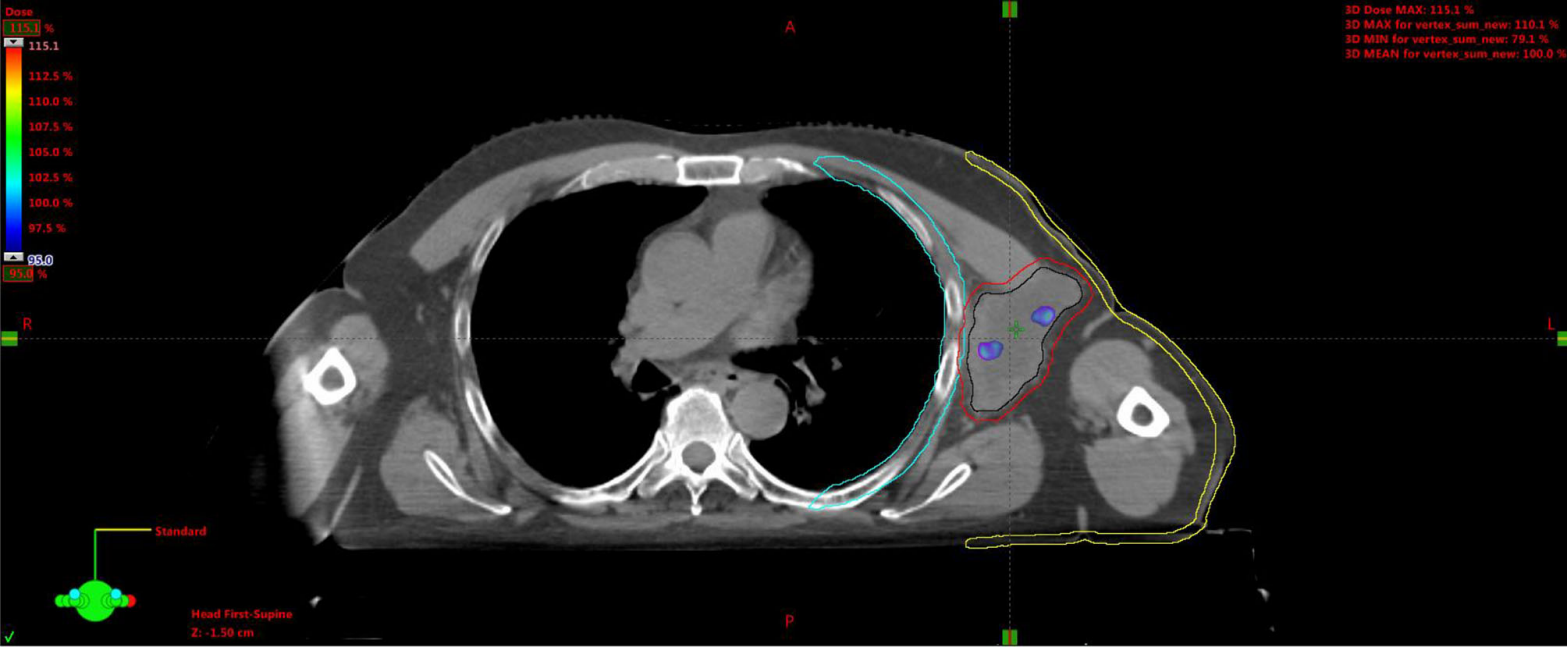
Plan D: 15 Gy to the vertices (red circles).

**Figure 11:**
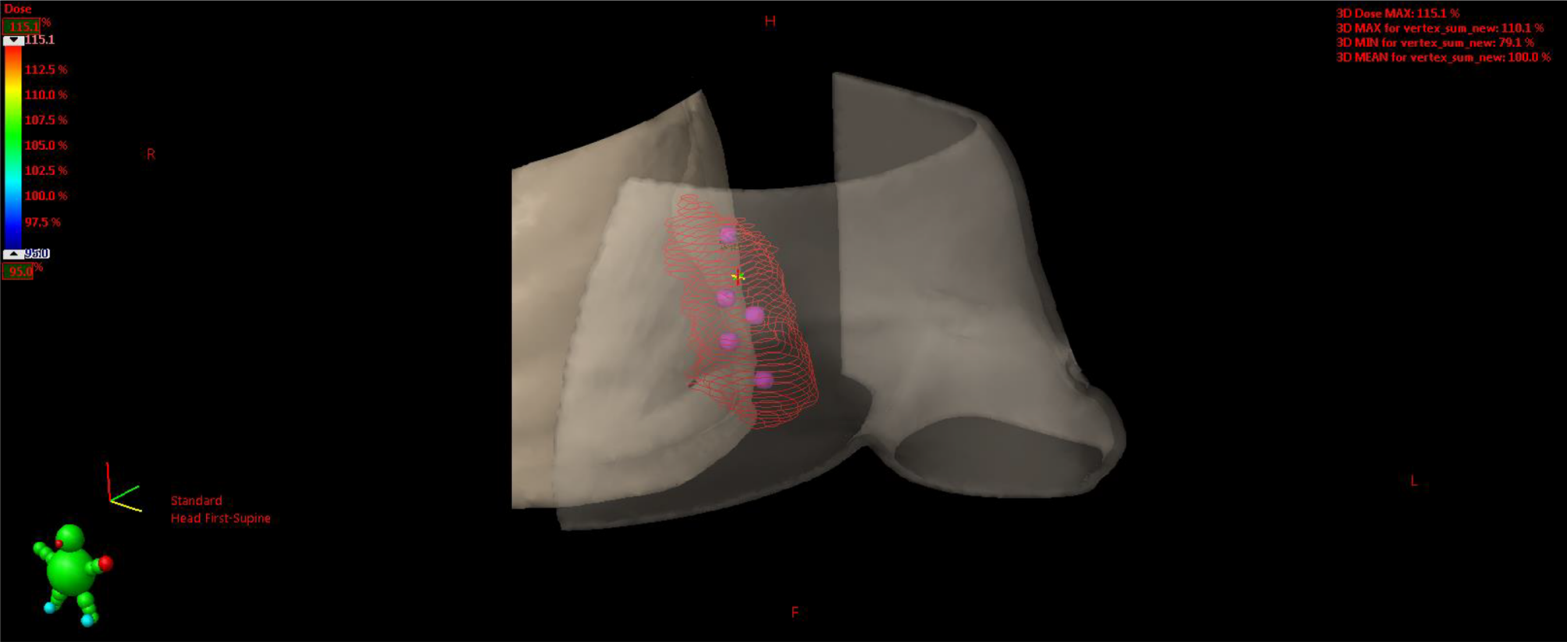
3D rendering of vertices within the GTV enclosed between the skin and chest wall. GTV, gross tumor volume.

The four plans were named as follows:

Plan A: 30 Gy in 10 daily fractions of 3 Gy each to the CTV (**Figure 7**), every time summed up to one of the following three.
Plan B: 15 Gy to the GTV in one fraction (**Figure 8**).
Plan C: 15 Gy to the hypoxic ring in one fraction (**Figure 9**).
Plan D: 15 Gy to the vertices in one shot (**Figures 10**, **11**).

For each plan, 2 half coplanar arcs (0°–179° CW/CCW) were used with jaw tracking technique, i.e., a specific technique that was provided by the Varian TrueBeam series (Varian, Crawley, UK), where the jaw can track the aperture of the MLC to reduce the leakage and transmission and thus reduce doses to normal tissues around the tumor.

For the other two disease sites, the above equipment and procedure were used for delivering an SBRT treatment with a dose of 30 Gy in five daily fractions of 6 Gy each.

The algorithm used for the plans was Anisotropic Analytical Algorithm (AAA version 13.7.14).

### Numerical Analysis

Computational analyses were performed in order to evaluate the ability of tumor control and the risk for OARs for the various plans considered.

In particular, two independent analyses were carried out: 1) calculation of the TCP using Poisson’s model (empirical model) and the NTCP adopting the LKB model; 2) *ad hoc* developed radiobiological model considering the different regions present within the tumor.

#### Tumor Control Probability and Normal Tissue Complication Probability Modeling

Structure sets and calculated three-dimensional (3D)-dose matrices of the RT plans were exported as DICOM files.

In order to calculate the TCP and NTCP values, the equivalent dose in 2 Gy per fraction, EQD_2_, was evaluated using the following expression:


$$EQD_{2}=D\left(\frac{d+\alpha /\beta }{2+\alpha /\beta }\right)$$

where D is the total dose in Gy, d is the dose per fraction, and *α*/*β* is the ratio of linear to quadratic cell killing probability according to the LQ model. In particular, for a tumor, the value of *α/β*=10.5Gy was used (18), and the values of *α/β*=8.8Gy (19) and *α/β*=3.5Gy (20) were adopted for the skin and chest wall, respectively. Since the spatial resolutions of the various plans considered were not identical, all plans were re-sampled to the highest resolution (1.5 × 1.5 × 1.5 mm^3^).

TCP was calculated based on the LQ Poisson’s model (21, 22) through the use of the pyradiobiology software (23, 24). The values of the parameters TCD50 and γ are those for head and neck squamous cells, at 51.77 Gy and 2.28, respectively (25).

For NTCP calculation for the skin and chest wall, the LKB model was applied (26). The parameters adopted for these calculations are n = 0.1, m = 0.21, and TD50 = 68.00 Gy for the case of pathological fracture of the chest wall and n = 0.1, m = 0.12, and TD50 = 70.00 Gy for the case of necrosis/ulceration of the skin (27).

#### *Ad Hoc* Radiobiological Model

The above-described analysis does not completely take into account the heterogeneity of the tumor region, and, therefore, the reliability of TCP values is limited. For this reason, a numerical radiobiological model able to consider various aspects of the complexity of the system was provided.

The proposed analytic method proposed to analyze the different radiobiological treatments is based on the following points:

- Tumor spheroid approximation since the volumes of the cell subpopulations are more relevant than their shapes;
- The radiation effect is described by the LQ model;
- The radioresistance of the hypoxic area is taken into account by the oxygen enhancement ratio (OER) approach;
- The vertices are localized in partial overlap with the necrotic and hypoxic volumes;
- The effects of the initial large dose (15 Gy) on the normoxic and hypoxic cells is described by average doses;
- 10 daily doses of 3 Gy follow the initial treatment.

The detail of the calculations is reported in **Supplementary Material 1**, and the final results compare (see *Discussion*) different methods of delivery of the large initial dose.

## Results

The effectiveness in tumor control and damage to healthy tissues was evaluated for the four different RT approaches by means of the two numerical studies mentioned above.

The analysis was performed *via* the pyradiobiology software, which calculates the TCP using Poisson’s model, and the NTCP adopting the LKB model. The results are reported in (**Table 1**).

**Table 1:** TCP values for tumor cells and NTCP values evaluated for the chest wall and skin.

	TCP Tumor	NTCP Chest Wall	NTCP Skin
Plan A	0.030	0.00040	2.0 * 10^−9^
Plan B	0.870	0.020	0.000015
Plan C	0.400	0.0076	0.000013
Plan D	0.032	0.00047	2.3 * 10^−9^

Plan A: 30 Gy/10 fractions to GTV. Plan B: 15 Gy/1 fraction to GTV. Plan C: 15 Gy/1 fraction to “hypoxic ring.” Plan D: 15 Gy/1 fraction to 5 vertices.

TCP, tumor control probability; NTCP, normal tissue complication probability; GTV, gross tumor volume.

From this analysis, it is evident that NTCP for the chest wall for Plan D (lattice) is comparable to that for Plan A (30 Gy in 10 fractions), and it is an order of magnitude smaller than that for Plan C (which is a combination of SBRT-PATHY as employed by Tubin et al. and a sequential palliative 30-Gy dose in 10 fractions of 3 Gy/day to the entire tumor volume) and 2 orders of magnitude smaller than that for Plan B (a single dose of 15 Gy homogeneously delivered to the entire tumor volume followed by 30 Gy in 10 fractions of 3 Gy/day).

Analogous behavior is observed for NTCP values related to skin exposure. Therefore, the lattice configuration of irradiation allows sparing healthy tissues better than hypoxic ring irradiation (Plan C) and entire volume irradiation (Plan B).

Regarding TCP analysis, Plan B is characterized by a high value (i.e., 87%), and this is related to the total volume irradiation with preliminary 15-Gy exposure, whereas in the case of Plan C (hypoxic ring irradiation), this probability is more than halved, and in the case of lattice irradiation (Plan D actually delivered to the patient), it is equal to about 3%. In order to consider also the heterogeneity of the tumor regions and to further model the response of this tumor to the RT procedure chosen, plans C and D were compared by means of an *ad hoc* developed radiobiological model, providing the respective cancer cell survival probability (CCSP) predictions. For such models, we used the α, β, and α/β values for cSCC suggested by van Leeuwen et al. (28). The numerical analysis in **Supplementary Material 1** carried out a detailed quantitative comparison between methods C and D, by evaluating the survival fraction before and after the standard treatment for the normoxic and hypoxic subpopulations. The results are reported in **Tables S2**, **S3** (**Supplementary Material 1**) where you can observe that the initial radiation dose of 15 Gy delivered to the whole hypoxic area (model C), rather than to the vertices (model D), gives a smaller survival probability, i.e., a better tumor control, but with a very large average dose (Dmean_normoxic_subvolume equal to 10.44 Gy for Plan C and to 4.3 Gy for Plan D) distributed in the nearby normoxic area.

## Discussion

### Background and Radiobiological Issues

This case report presents a novel technique to treat bulky tumors, generally treated with palliative-only RT. The peculiarity of the RT technique here presented is mainly linked to the ability to deliver high radiation doses to small areas of the GTV, so that radiation oncologists can treat with a higher total dose the GTV as compared to other techniques using lower homogeneous radiation doses due to the close proximity of OARs. Furthermore, as regards the treatment planning, this is no more difficult than the other volumetric plans; it is likely more time-consuming because the radiation oncologist has to choose the vertex positioning within the GTV and to co-register PET/CT images with simulation CT images. Finally, the time to deliver radiation doses to the vertices is no different than that of other modulated/stereotactic techniques. The palliative radiation approach could not achieve a satisfying TCP, thus adversely affecting the patient survival chances. This issue is particularly relevant when a large mass develops in the context of an overall limited tumor burden, as in the present case, where it is reasonable to expect a better outcome than a multimetastatic setting (29–32). Currently, the oligometastatic disease is effectively treated by stereotactic RT, that is, with an approximately homogeneous radiation dose delivered to the entire tumor volume (33). Such an approach would appear to allow a deferral in the use of aggressive chemotherapy regimens even among those patients affected by oligometastatic mucosal HNSCC (34). The advanced and/or metastatic cSCC counterpart may be treated with targeted therapies, but such a consideration does not rule out a key role for ablative RT (35, 36), not yet extensively tested due to the low rate of metastatic disease (37). However, bulky masses could not be treated with high-dose stereotactic RT without exceeding the normal tissue tolerance (38). Alternative solutions to deliver ablative radiation doses are under study, and SFRT is among them (39). Such a method is supposed to trigger a killing bystander effect (**Figure 2**) on the underdosed tumor subvolumes (40). Bulky tumors are characterized by a heterogeneous oxygen supply that generates an alternation of well-oxygenated proliferating areas and hypoxic “dormant” areas. Notoriously, tumor hypoxia represents the main obstacle to the full effectiveness of RT (41). For a given dose, such a condition increases the cancer cell survival fraction both *in vitro* and *in vivo*. This issue may occur for two reasons: 1) due to a spatial limit of oxygen diffusion to cells that are more distantly located from newly formed vessels during disordered neoangiogenic sprouting or 2) to a transient mechanic occlusion of such capillaries because of endothelial cell abnormalities, starving harder the innermost cells. Both phenomena determine a significant reduction in the partial pressure of oxygen (pO₂) that, for the aforesaid reasons, is inhomogeneous within the tumor tissue. Considering that the oxygen path is mostly stopped at a depth from the vessel of 100–200 μm, it explains how, beyond this threshold, cell cascades culminating in necrosis may be triggered (42). Thus, it is not infrequent to find a necrotic core surrounded by a vital cell ring. It is reasonable to assume that between normoxic and necrotic areas, hypoxic cell clones exist. These cells, for example, through—but not only—the hypoxia-inducible factor 1α (HIF-1α) signaling pathway, develop a metabolic adaptation to hypoxia and survive (43). They may be not actively proliferating but still viable and able to escape from the radiation effects. In fact, oxygen enhances free radical formation and fixes chemical damages induced by ionizing radiations, especially double-strand DNA breaks. The variability of cell survival rate at different levels of pO₂ is summarized by the OER parameter. Therefore, oxygen deprivation, by increasing tumor cell survival, could impair oncologic outcomes, such as local control (LC) and, consequently, also overall survival (OS). Indeed, the clearance of normoxic cells by radiation could recruit previously hypoxic cells to be exposed to a better promitotic pO₂, thus enhancing their proliferative and metastatic potential. Such cell behavior is at the root of radiation dose fractionation in clinical practice. In fact, the tumor redox landscape constantly changes in parallel with variations in cellular density (44). However, total removal of all cells is not always achievable. This applies especially to bulky tumors whose absolute number of hypoxic cells may be very high. In this scenario, another issue becomes evident: as expected, the unintended dose delivered to nearby healthy tissues (OARs) increases when the target volume increases due to a deleterious dose–volume effect (45). Then, radiation oncologists face daily this dose-limiting factor associated with a poor radiosensitivity of the hypoxic components. To counteract the depletion of tumor oxygen, various strategies such as the use of radiosensitizers have been tested to improve the therapeutic ratio of radiation dose. Unfortunately, practical results have not always confirmed the theoretical assumptions, likely because the topic is even more complex (46). Regarding the dose fraction size, it is known that large ones could overcome hypoxia-related radioresistance, but these could not be easily deliverable in a uniform manner to the entire tumor due to the aforementioned dose–volume effect that puts a strain on the tolerance of neighboring OARs. A compromise solution could be to deliver a high radiation dose/fraction solely in some tumor subvolumes, namely, the hypoxic ones. This is the actual basis for oxygen-guided RT. This novel concept is based on the detection of hypoxic subvolumes within tumor tissue for a selective radiation boost while limiting the unnecessarily escalated dose to well-oxygenated and radiosensitive ones. Such a pattern of radiation dose delivery is shared with another treatment strategy, the SFRT, basically known in two main forms: the GRID and the lattice one (39). These two differ by a more rigorously geometric arrangement of high-dose regions in the first respect to the second one. Both treatments were created as non-oxygen guided. We believe that an SFRT technique based on hypoxia tracking could enhance the synergy between high radiation dose, able to eradicate hypoxic clones, and the subsequently elicited radiobiological effects, which could promote tumor regression also in low-dose regions as well as distantly in non-targeted lesions (11). However, cell interactions against the background of the host immune system are not fully known and, consequently, not purposely engageable to lead an antitumor response. On the other hand, by adopting an *in silico* model to explain the interaction (that is, the killing effect) between radiation dose and hypoxic cells, the radical dose to eliminate them all, even after taking into account the time between a fraction and the next one, could be approximately predicted (12).

### Clinical Implications and Perspectives

In clinical practice, the finding of bulky tumors is not uncommon. A large part of them sometimes presents a necrotic core, detectable as a deeper hypodense region on CT scans (47, 48) or as photopenic on ^18^F-FDG PET images (49). The most common approach used in these cases, especially when these masses are metastases, is to treat the total volume with a homogeneous palliative dose, so as not to exceed the tolerance of OARs at the boundary with the target periphery. Clearly, such a strategy is unable to produce a durable LC and, subsequently, could address the need for re-treatment, obviously not without serious concerns regarding toxicities. Conversely, a uniform high-dose delivery could be not deliverable without a hazardous dose–volume effect. Hence, the radiation oncologist is often forced to settle for the first option. To circumvent these risks, recently, Tubin et al. proposed an unconventional irradiation technique for partially treating inhomogeneous bulky tumors (SBRT-PATHY): to deliver a high radiation dose to hypoxic areas with a sharp dose fall-off toward the outside of the tumor in order to spare the normoxic portion and, above all, the peripheral microenvironment for evoking immune radiobiological effects (bystander and abscopal) (50). This strategy achieved great clinical results in terms of tumor volume regression with no additional toxicity as compared to conventional palliative treatment (16). In this technique, the low-dose region represented by the normoxic peripheral tumor ring acts as a “buffer” between the high-dose hypoxic rim and the adjacent OARs to spare. However, further clinical trials with a larger sample size are needed prior to translating these preliminary results to routine clinical practice. In fact, partially uncovering some tumor subvolumes could be at least controversial or even hazardous from the radiation oncologist’s point of view. The concern about the difficulty in precisely mapping the hypoxic areas for selective irradiation could deter the implementation of this strategy. After all, the spatial resolution of current clinical imaging could be unable to detect all minor hypoxic regions. The consequence of missing out on the required radiation dose to one of these areas may be poor tumor control. For this reason, an alternative to SBRT-PATHY could be to selectively boost detectable hypoxic areas while maintaining a low effective dose in the remaining normoxic areas. Such an approach could balance the need for a booster dose in radioresistant hypoxic regions with the need to cover the overall tumor volume with an adequate radiation dose to hamper a rapid tumor regrowth while preserving nearby normal tissues. This strategy is calling for a mathematical model that can help to determine the required radiation dose to overcome hypoxic radioresistance. For this purpose, we have chosen a single dose of 15 Gy because this dose size has proven to be more effective in terms of tumor regression when compared to lower doses (51). Moreover, the single shot allowed us to bypass the redistribution in tumor oxygenation, following each radiation dose in fractionated schedules. In fact, the tumor oxygen map constantly evolves after radiation due to the recovery from vaso-occlusive events in tumor vessels on the one hand and to the prothrombotic effect associated with the swelling of irradiated tumor endothelial cells on the other (52). Among the non-invasive techniques currently available for measuring oxygen levels in tumor tissues, we find optical methods, those based on NMR, and nuclear medicine techniques. PET/CT has several advantages: a good intrinsic resolution, a 3D representation of the tumor, the possibility of making semiquantitative evaluations of the hypoxic tumor load, the ease of execution, the reproducibility of the data, and above all the highest diagnostic specificity in the characterization of the hypoxic tissue (53). A large number of PET tracers have now been developed for the identification of hypoxia. ^18^F-fluoromisonidazole is the most studied PET tracer for hypoxia imaging, but the accumulation of the tracer in the hypoxic tissues is rather slow, and the tumor/background ratio is still quite low. Therefore, only a few radiopharmacies produce specific tracers for hypoxia, and procurement is very difficult. ^18^F-FDG is the most widely used PET tracer in oncology, as cancer cells usually show an increase in their metabolism and in particular in glycolysis, even in conditions of aerobiosis (Warburg effect). The increase in the expression of glucose transporters and glycolytic enzymes can also be activated by hypoxia, through the factor HIF-1α (anaerobic glycolysis, the Pasteur effect) (53). Apart from the constitutive photopenia of large tumor necrosis, the uptake of FDG under both normal and reduced oxygen pressure conditions obviously makes FDG non-specific for hypoxia. However, FDG-PET/CT proved to be a good tool in staging patients with cSCC as well (54). Other studies have reported nodal involvement sensitivities ranging between 91% and 100% and change in management in 6.25%–40% of patients (55–57). A recent study in node-positive cSCC patients calculated the positive and negative predictive values for nodal detection in preoperative FDG-PET/CT as 91.1% and 66.7%, respectively (56). As a consequence of this, the use of such an imaging to study and characterize the tumor disease in our patient was appropriate.

### Mathematical Radiobiological Models: A Call For New Ones?

Taking into account the different dose prescriptions and a more and more detrimental dose–volume effect as the irradiated volume increases, Plan A would have an excellent NTCP but the worst TCP. Conversely, Plan B would be likely characterized by an excellent TCP and a not negligible NTCP for the chest wall (2%) when compared to plans C and D (58) and at the expense of the target dose coverage as evidenced by the cumulative DVH shown in **Figure S1** of **Supplementary Material 2** (see the smoother slope of the orange curve nearby the dose prescription value). As these outcomes do not fit a radical and safe curative purpose, we developed an *ad hoc* mathematical radiobiological model only for plans C and D in order to further investigate their respective CCSPs. Interestingly, the numerical results about the cell survival rate according to the LQ model show a strong reduction of the subpopulation volumes, although with a TCP that could be small for a large clonogenic number (see **Tables S2**, **S3** on the cell survival fraction). This means that the LQ model could not be able to explain the reported cCR after lattice RT delivery. Such a finding supports our assumption about immune intervention even more. After all, the involvement of immune response, specifically the recruitment of effector immune cells at the distant disease sites, needed to be adduced to explain the abscopal effect reported in a case with multiple nodules of cSCC, of which only one was irradiated (59). As Plan A employed a palliative dose by definition and Plan B a not safely deliverable uniform high dose, respectively, we deemed it useless to provide CCSP for them. Conversely, we elaborated NTCP for the skin and chest wall for all four dose prescriptions, purposely rescaled in EQD2 (Equivalent Dose in 2 Gy/Fx) value. As evidenced by cumulative EQD2-DVHs in **Supplementary Material 2**, the volume of the skin and chest wall for Plan D was constantly lower than that for Plans B and C at each dose level. In other terms, the addition of vertices dose has an almost negligible effect over the two OARs when compared to the other two plans. This was particularly evident at the highest dose levels, as inferred from the differential DVHs. However, all plans have a very-low near-zero risk of damage for both the chest wall and skin. Indeed, as a more detailed examination shows, the near-zero risk of Plan D is of another order of magnitude in comparison with that of Plans B and C, e.g., up to 10,000 times smaller for the skin. In fact, on the NTCP curve, the value of risk for Plan D is just beyond that of Plan A and significantly distant from that of Plans B and C, which, conversely, are closer to the curve section with the greatest slope that indicates a very real danger for the chest wall and skin. Also regarding the chest wall, the largest volume to be boosted in Plan B entails a low but significant risk (≈2%) when compared to the risks of Plans C and D. This means that Plan D is a more precautionary method of radiation delivery. Such a finding could be mainly useful in case of the need for re-treatment or treatment of neighboring metachronous metastases. Moreover, it has to be emphasized that NTCP predictions are largely influenced by the amount of OAR volume under investigation. We considered a very large portion of the skin and chest wall, namely, the one approximately exposed to at least 2 Gy because no dose is without sequelae. Thus, in the NTCP simulation, the risk prediction related to the highest doses could be underrated if such doses are computed as scattered in a very large volume rather than as concentrated in a small one. Starting from these considerations, the lattice approach could be even more advantageous than it appears in this particular case. The flexibility for high-dose vertices positioning permits to maximize tumor control without threatening the surrounding healthy tissues. This assumption can be valid also for other tumor histology and high-risk locations. Of particular note, the lattice dose delivery permitted a more rapid tumor response than the other two disease sites treated with classic stereotactic RT.

### Advantages From This Preliminary Experience

As cSCC is prevalent among very elderly and frail patients, our SFRT solution could be valuable also for the treatment of primary bulky lesions in those cases not eligible for radical surgery due to a high anesthetic risk or technical complexity (60). In this patient setting, the treatment option here presented could be safer and more tolerable than the multi-drug systemic therapies investigated in currently ongoing trials (61). Besides, such immuno- and chemotherapeutic agents may struggle to penetrate within hypo-vascularized hypoxic tumor subdomains with a consequent decline in LC. Also, well-established drug protocols, like the one here used, based on cemiplimab, are not without serious concerns about toxicities and still have a poor overall response rate (62). To improve the latter, a combination of cemiplimab with hypofractionated RT has been already tested with encouraging results (63).

### Limitations

We are fully aware that this study has several limitations. First of all, one swallow does not make a summer: a case report does not allow to draw any definitive conclusions. Secondly, all the numerical analyses are based on the estimated number of cells contained in each voxel, in a way that their computation is, of course, largely approximate. Thirdly, the failure to use clinical imaging that was specifically devoted to accurately distinguishing the different subvolumes (normoxic, hypoxic, and necrotic ones) represents another weakness. In any case, in this regard, on the basis of ^18^F-FDG tracer distribution, it is reasonable to assume that a transitional layer populated by oxygen-starved cells rings an innermost photopenic area due to necrosis. Fourthly, the whole procedure of target contouring, particularly the choice of vertex number, size, dose, and positioning, was strictly operator-dependent and this could undermine the reproducibility of the result. Fifthly, the role of lattice RT in achieving the reported results may be overestimated due to the use of cemiplimab following the RT course, although it must be emphasized that the cCR in the bulky axillary lesion was documented already prior to the drug administration. However, we did not just present our successful approach but even tried to provide a reliable scientific insight, in agreement with the currently available mathematical radiobiological models. It is indeed worth stressing that the LQ model predicted a very small value of TCP for the delivered plan (3%), but nevertheless, we observed a very positive outcome: actually, there is the possibility that we described just one of the three cases out of 100 with tumor control. Further investigations on large series with similar tumors should be performed in order to confirm the reliability of this model in describing this type of RT planning.

### Final Considerations

To the best of our knowledge, this is the first report about lattice RT in which the vertex positioning was based on the ^18^F-FDG PET-detected metabolic heterogeneity within a large tumor mass as a surrogate of its oxygen landscape and whose clinical outcome was attempted to be explained either by the currently available mathematical radiobiological models or an *ad hoc* developed one. Neither the first nor the second could be sufficient to exhaustively explain the reported outcome, likely calling for more complex radiobiological models.

The list of successful lattice RT applications now includes bulky cSCCs.

## Conclusions

Lattice RT might be safe and effective for the treatment of bulky locally advanced or metastatic cSCCs. Large trials are needed to draw up tailored RT for difficult-to-treat cancer patients, and wide investigations are needed to deeply evaluate the effectiveness of the lattice RT. Moreover, more theoretical analyses with existent and/or novel radiobiological models could be useful to explain lattice RT effects, also taking into account the reaction of the host immune system to this particular radiation dose delivery.

## Data Availability Statement

The original contributions presented in the study are included in the article/**Supplementary Material**. Further inquiries can be directed to the corresponding author.

## Ethics Statement

Ethical approval was not provided for this study on human participants because ethical approval was waived in view of the retrospective nature of the study, and all the procedures being performed were part of the routine care. This research study was conducted retrospectively from data obtained for clinical purposes and is in accordance with the local legislation and institutional requirements. The patients/participants provided their written informed consent to participate in this study.

## Author Contributions

All authors contributed equally to the paper. GF and PC shared the first authorship. MM and SP shared the last authorship.

## Conflict of Interest

IS is co-founder of the company Medical Innovation and Technology P.C.

The remaining authors declare that the research was conducted in the absence of any commercial or financial relationships that could be construed as a potential conflict of interest.

## Publisher’s Note

All claims expressed in this article are solely those of the authors and do not necessarily represent those of their affiliated organizations, or those of the publisher, the editors and the reviewers. Any product that may be evaluated in this article, or claim that may be made by its manufacturer, is not guaranteed or endorsed by the publisher.

## References

[1] Ferini G Molino L Bottalico L De Lucia P Garofalo F. A Small Case Series About Safety and Effectiveness of a Hypofractionated Electron Beam Radiotherapy Schedule in Five Fractions for Facial Non Melanoma Skin Cancer Among Frail and Elderly Patients. Rep Pract Oncol Radiother (2021) 26(1):66–72. doi: 10.5603/RPOR.a2021.0013 33948304 PMC8086708

[2] Cherpelis BS Marcusen C Lang PG. Prognostic Factors for Metastasis in Squamous Cell Carcinoma of the Skin. Dermatol Surg (2002) 28(3):268–73. doi: 10.1046/j.1524-4725.2002.01169.x 11896781

[3] Vincent AG Wang W Shokri T Ducic Y. Treatment of Oligometastatic Disease in Squamous Cell Carcinoma of the Head and Neck. Laryngoscope (2021) 131(5):E1476–80. doi: 10.1002/lary.29115 PMC8246782 33044014

[4] Green AC Olsen CM. Cutaneous Squamous Cell Carcinoma: An Epidemiological Review. Br J Dermatol (2017) 177(2):373–81. doi: 10.1111/bjd.15324 28211039

[5] AIRTUM Working Group Busco S Buzzoni C Mallone S Trama A Castaing M. Italian Cancer Figures–Report 2015: The Burden of Rare Cancers in Italy. Epidemiol Prev (2016) 40(1 Suppl 2):1–120. doi: 10.19191/EP16.1S2.P001.035 26951748

[6] Available at: https://www.nccn.org/professionals/physician_gls/pdf/squamous.pdf.

[7] Schmults CD Karia PS Carter JB Han J Qureshi AA. Factors Predictive of Recurrence and Death From Cutaneous Squamous Cell Carcinoma: A 10-Year, Single-Institution Cohort Study. JAMA Dermatol (2013) 149(5):541–7. doi: 10.1001/jamadermatol.2013.2139 23677079

[8] Cacciola A Parisi S Tamburella C Lillo S Ferini G Molino L. Stereotactic Body Radiation Therapy and Radiofrequency Ablation for the Treatment of Liver Metastases: How and When? Rep Pract Oncol Radiother (2020) 25(3):299–306. doi: 10.1016/j.rpor.2020.02.010 32194349 PMC7078501

[9] Pontoriero A Iatì G Cacciola A Conti A Brogna A Siragusa C. Stereotactic Body Radiation Therapy With Simultaneous Integrated Boost in Patients With Spinal Metastases. Technol Cancer Res Treat (2020) 19:1533033820904447. doi: 10.1177/1533033820904447 32336255 PMC7225842

[10] D’Angelillo RM Ingrosso G Ravo V Triggiani L Magli A Mazzeo E. Consensus Statements on Ablative Radiotherapy for Oligometastatic Prostate Cancer: A Position Paper of Italian Association of Radiotherapy and Clinical Oncology (AIRO). Crit Rev Oncol Hematol (2019) 138:24–8. doi: 10.1016/j.critrevonc.2019.03.014 31092381

[11] Ferini G Valenti V Tripoli A Illari SI Molino L Parisi S. Lattice or Oxygen-Guided Radiotherapy: What If They Converge? Possible Future Directions in the Era of Immunotherapy. Cancers (Basel) (2021) 13(13):3290. doi: 10.3390/cancers13133290 34209192 PMC8268715

[12] Castorina P Castorina L Ferini G. Non-Homogeneous Tumor Growth and Its Implications for Radiotherapy: A Phenomenological Approach. J Pers Med (2021) 11(6):527. doi: 10.3390/jpm11060527 34207503 PMC8229245

[13] Warkentin B Stavrev P Stavreva N Field C Fallone BG. A TCP-NTCP Estimation Module Using DVHs and Known Radiobiological Models and Parameter Sets. J Appl Clin Med Phys (2004) 5(1):50–63. doi: 10.1120/jacmp.v5i1.1970 15753933 PMC5723441

[14] Vadalà RE Santacaterina A Sindoni A Platania A Arcudi A Ferini G. Stereotactic Body Radiotherapy in Non-Operable Lung Cancer Patients. Clin Transl Oncol (2016) 18(11):1158–9. doi: 10.1007/s12094-016-1552-7 27686231

[15] Bentzen SM Joiner MC. The Linear-Quadratic Approach in Clinical Practice. In: Joiner MC van der Kogel A, editors. Basic Clin. Radiobiol, 4th ed. London: Hodder Arnold (2009).

[16] Tubin S Khan MK Salerno G Mourad WF Yan W Jeremic B. Mono-Institutional Phase 2 Study of Innovative Stereotactic Body RadioTherapy Targeting PArtial Tumor HYpoxic (SBRT-PATHY) Clonogenic Cells in Unresectable Bulky Non-Small Cell Lung Cancer: Profound Non-Targeted Effects by Sparing Peri-Tumoral Immune Microenvironment. Radiat Oncol (2019) 14(1):212. doi: 10.1186/s13014-019-1410-1 31771654 PMC6878646

[17] Kishikawa T Suzuki M Takemoto N Fukusumi T Michiba T Hanamoto A. Response Evaluation Criteria in Solid Tumors (RECIST) and PET Response Criteria in Solid Tumors (PERCIST) for Response Evaluation of the Neck After Chemoradiotherapy in Head and Neck Squamous Cell Carcinoma. Head Neck (2021) 43(4):1184–93. doi: 10.1002/hed.26583 33368784

[18] Stuschke M Thames HD. Fractionation Sensitivities and Dose-Control Relations of Head and Neck Carcinomas: Analysis of the Randomized Hyperfractionation Trials. Radiother Oncol (1999) 51(2):113–21. doi: 10.1016/s0167-8140(99)00042-0 10435801

[19] Turesson I Thames HD. Repair Capacity and Kinetics of Human Skin During Fractionated Radiotherapy: Erythema, Desquamation, and Telangiectasia After 3 and 5 Year’s Follow-Up. Radiother Oncol (1989) 15(2):169–88. doi: 10.1016/0167-8140(89)90131-x 2762590

[20] Bentzen SM Overgaard M Thames HD. Fractionation Sensitivity of a Functional Endpoint: Impaired Shoulder Movement After Post-Mastectomy Radiotherapy. Int J Radiat Oncol Biol Phys (1989) 17(3):531–7. doi: 10.1016/0360-3016(89)90103-x 2506157

[21] Munro TR Gilbert CW. The Relation Between Tumour Lethal Doses and the Radiosensitivity of Tumour Cells. Br J Radiol (1961) 34:246–51. doi: 10.1259/0007-1285-34-400-246 13726846

[22] Brahme A Agren AK. Optimal Dose Distribution for Eradication of Heterogeneous Tumours. Acta Oncol (1987) 26(5):377–85. doi: 10.3109/02841868709104364 3426851

[23] Available at: https://www.sachpazidis.com/software-projects/pyradiobiology/.

[24] Spohn SKB Sachpazidis I Wiehle R Thomann B Sigle A Bronsert P. Influence of Urethra Sparing on Tumor Control Probability and Normal Tissue Complication Probability in Focal Dose Escalated Hypofractionated Radiotherapy: A Planning Study Based on Histopathology Reference. Front Oncol (2021) 11:652678. doi: 10.3389/fonc.2021.652678 34055621 PMC8160377

[25] Okunieff P Morgan D Niemierko A Suit HD. Radiation Dose-Response of Human Tumors. Int J Radiat Oncol Biol Phys (1995) 32(4):1227–37. doi: 10.1016/0360-3016(94)00475-z 7607946

[26] Semenenko VA Li XA. Lyman-Kutcher-Burman NTCP Model Parameters for Radiation Pneumonitis and Xerostomia Based on Combined Analysis of Published Clinical Data. Phys Med Biol (2008) 53(3):737–55. doi: 10.1088/0031-9155/53/3/014 18199912

[27] Emami B Lyman J Brown A Coia L Goitein M Munzenrider JE. Tolerance of Normal Tissue to Therapeutic Irradiation. Int J Radiat Oncol Biol Phys (1991) 21(1):109–22. doi: 10.1016/0360-3016(91)90171-y 2032882

[28] van Leeuwen CM Oei AL Crezee J Bel A Franken NAP Stalpers LJA. The Alfa and Beta of Tumours: A Review of Parameters of the Linear-Quadratic Model, Derived From Clinical Radiotherapy Studies. Radiat Oncol (2018) 13(1):96. doi: 10.1186/s13014-018-1040-z 29769103 PMC5956964

[29] Zhang Y Schoenhals J Christie A Mohamad O Wang C Bowman I. Stereotactic Ablative Radiation Therapy (SAbR) Used to Defer Systemic Therapy in Oligometastatic Renal Cell Cancer. Int J Radiat Oncol Biol Phys (2019) 105(2):367–75. doi: 10.1016/j.ijrobp.2019.07.023 PMC7647381 31377159

[30] Zhang B Leech M. A Review of Stereotactic Body Radiation Therapy in the Management of Oligometastatic Prostate Cancer. Anticancer Res (2020) 40(5):2419–28. doi: 10.21873/anticanres.14211 32366385

[31] Zhao Y Li J Li D Wang Z Zhao J Wu X. Tumor Biology and Multidisciplinary Strategies of Oligometastasis in Gastrointestinal Cancers. Semin Cancer Biol (2020) 60:334–43. doi: 10.1016/j.semcancer.2019.08.026 31445220

[32] Zhou Y Yu F Zhao Y Zeng Y Yang X Chu L. A Narrative Review of Evolving Roles of Radiotherapy in Advanced Non-Small Cell Lung Cancer: From Palliative Care to Active Player. Transl Lung Cancer Res (2020) 9(6):2479–93. doi: 10.21037/tlcr-20-1145 PMC7815368 33489808

[33] Zayed S Correa RJM Palma DA. Radiation in the Treatment of Oligometastatic and Oligoprogressive Disease: Rationale, Recent Data, and Research Questions. Cancer J (2020) 26(2):156–65. doi: 10.1097/PPO.0000000000000436 32205541

[34] Bonomo P Greto D Desideri I Loi M Di Cataldo V Orlandi E. Clinical Outcome of Stereotactic Body Radiotherapy for Lung-Only Oligometastatic Head and Neck Squamous Cell Carcinoma: Is the Deferral of Systemic Therapy a Potential Goal? Oral Oncol (2019) 93:1–7. doi: 10.1016/j.oraloncology.2019.04.006 31109688

[35] Zhou J Liu R Luo C Zhou X Xia K Chen X. MiR-20a Inhibits Cutaneous Squamous Cell Carcinoma Metastasis and Proliferation by Directly Targeting LIMK1. Cancer Biol Ther (2014) 15(10):1340–9. doi: 10.4161/cbt.29821 PMC4130727 25019203

[36] Zilberg C Lee MW Kraitsek S Ashford B Ranson M Shannon K. Is High-Risk Cutaneous Squamous Cell Carcinoma of the Head and Neck a Suitable Candidate for Current Targeted Therapies? J Clin Pathol (2020) 73(1):17–22. doi: 10.1136/jclinpath-2019-206038 31300530

[37] Karia PS Han J Schmults CD. Cutaneous Squamous Cell Carcinoma: Estimated Incidence of Disease, Nodal Metastasis, and Deaths From Disease in the United States, 2012. J Am Acad Dermatol (2013) 68(6):957–66. doi: 10.1016/j.jaad.2012.11.037 23375456

[38] Bujold A Massey CA Kim JJ Brierley J Cho C Wong RK. Sequential Phase I and II Trials of Stereotactic Body Radiotherapy for Locally Advanced Hepatocellular Carcinoma. J Clin Oncol (2013) 31(13):1631–9. doi: 10.1200/JCO.2012.44.1659 23547075

[39] Billena C Khan AJ. A Current Review of Spatial Fractionation: Back to the Future? Int J Radiat Oncol Biol Phys (2019) 104(1):177–87. doi: 10.1016/j.ijrobp.2019.01.073 PMC7443362 30684666

[40] Pellizzon ACA. Lattice Radiation Therapy - Its Concept and Impact in the Immunomodulation Cancer Treatment Era. Rev Assoc Med Bras (1992) (2020) 66(6):728–31. doi: 10.1590/1806-9282.66.6.728 32696876

[41] Rockwell S Dobrucki IT Kim EY Marrison ST Vu VT. Hypoxia and Radiation Therapy: Past History, Ongoing Research, and Future Promise. Curr Mol Med (2009) 9(4):442–58. doi: 10.2174/156652409788167087 PMC2752413 19519402

[42] Small W. Perez and Brady’s Principles and Practice of Radiation Oncology. JAMA (2009) 301:2046. doi: 10.1001/jama.2009.718

[43] Lee SH Golinska M Griffiths JR. HIF-1-Independent Mechanisms Regulating Metabolic Adaptation in Hypoxic Cancer Cells. Cells (2021) 10(9):2371. doi: 10.3390/cells10092371 34572020 PMC8472468

[44] Hall EJ Giaccia AJ. Radiobiology for the Radiologist. Philadelphia, PA, USA: Wolters Kluwer Health/Lippincott Williams & Wilkins (2012).

[45] Li Q Chen J Zhu B Jiang M Liu W Lu E. Dose Volume Effect of Acute Diarrhea in Post-Operative Radiation for Gynecologic Cancer. Rev Invest Clin (2017) 69(6):329–35. doi: 10.24875/RIC.17002373 29265117

[46] Eschwège F Sancho-Garnier H Chassagne D Brisgand D Guerra M Malaise EP. Results of a European Randomized Trial of Etanidazole Combined With Radiotherapy in Head and Neck Carcinomas. Int J Radiat Oncol Biol Phys (1997) 39(2):275–81. doi: 10.1016/s0360-3016(97)00327-1 9308928

[47] Miyake H Hori Y Dono S Mori H. Low Attenuation Intratumoral Matrix: CT and Pathologic Correlation. J Comput Assist Tomogr (2000) 24(5):761–72. doi: 10.1097/00004728-200009000-00018 11045700

[48] Shan X Wang D Chen J Xiao X Jiang Y Wang Y. Necrosis Degree Displayed in Computed Tomography Images Correlated With Hypoxia and Angiogenesis in Breast Cancer. J Comput Assist Tomogr (2013) 37(1):22–8. doi: 10.1097/RCT.0b013e318279abd1 23321829

[49] Ashley Cox R Akhurst T Bressel M MacManus M Ball D. Survival and Central Photopenia Detected by Fluorine-18 Fluoro-Deoxy-Glucose Positron Emission Tomography (FDG-PET) in Patients With Locoregional Non-Small Cell Lung Cancer Treated With Radiotherapy. Radiother Oncol (2017) 124(1):25–30. doi: 10.1016/j.radonc.2017.06.004 28662870

[50] Tubin S Popper HH Brcic L. Novel Stereotactic Body Radiation Therapy (SBRT)-Based Partial Tumor Irradiation Targeting Hypoxic Segment of Bulky Tumors (SBRT-PATHY): Improvement of the Radiotherapy Outcome by Exploiting the Bystander and Abscopal Effects. Radiat Oncol (2019) 14(1):21. doi: 10.1186/s13014-019-1227-y 30696472 PMC6352381

[51] Mohiuddin M Fujita M Regine WF Megooni AS Ibbott GS Ahmed MM. High-Dose Spatially-Fractionated Radiation (GRID): A New Paradigm in the Management of Advanced Cancers. Int J Radiat Oncol Biol Phys (1999) 45(3):721–7. doi: 10.1016/s0360-3016(99)00170-4 10524428

[52] Brat DJ Van Meir EG. Vaso-Occlusive and Prothrombotic Mechanisms Associated With Tumor Hypoxia, Necrosis, and Accelerated Growth in Glioblastoma. Lab Invest (2004) 84(4):397–405. doi: 10.1038/labinvest.3700070 14990981

[53] Lopci E Grassi I Chiti A Nanni C Cicoria G Toschi L. PET Radiopharmaceuticals for Imaging of Tumor Hypoxia: A Review of the Evidence. Am J Nucl Med Mol Imaging (2014) 4(4):365–84. PMC4074502 24982822

[54] Mahajan S Barker CA Sing B Pandit-Taskar N. Clinical Value of 18F-FDG-PET/CT in Staging Cutaneous Squamous Cell Carcinoma. Nucl Med Commun (2019) 40(7):744–51. doi: 10.1097/MNM.0000000000001029 PMC7518232 31095044

[55] Fujiwara M Suzuki T Takiguchi T Fukamizu H Tokura Y. Evaluation of Positron Emission Tomography Imaging to Detect Lymph Node Metastases in Patients With High-Risk Cutaneous Squamous Cell Carcinoma. J Dermatol (2016) 43:1314–20. doi: 10.1111/1346-8138.13403 27060693

[56] Hirshoren N Olayos E Herschtal A Kumar ASR Gyorki DE. Preoperative Positron Emission Tomography for Node-Positive Head and Neck Cutaneous Squamous Cell Carcinoma. Otolaryngol Head Neck Surg (2018) 158:122–6. doi: 10.1177/0194599817731735 28925330

[57] Supriya M Suat-Chin N Sizeland A. Use of Positron Emission Tomography Scanning in Metastatic Head and Neck Cutaneous Squamous Cell Cancer: Does it Add to Patient Management? Am J Otolaryngol (2014) 35:347–52. doi: 10.1016/j.amjoto.2014.01.006 24503246

[58] Bongers EM Haasbeek CJ Lagerwaard FJ Slotman BJ Senan S. Incidence and Risk Factors for Chest Wall Toxicity After Risk-Adapted Stereotactic Radiotherapy for Early-Stage Lung Cancer. J Thorac Oncol (2011) 6(12):2052–7. doi: 10.1097/JTO.0b013e3182307e74 22052227

[59] Bellia SR Feliciani G Duca MD Monti M Turri V Sarnelli A. Clinical Evidence of Abscopal Effect in Cutaneous Squamous Cell Carcinoma Treated With Diffusing Alpha Emitters Radiation Therapy: A Case Report. J Contemp Brachytherapy (2019) 11(5):449–57. doi: 10.5114/jcb.2019.88138 PMC6854861 31749854

[60] Lupon E Lellouch AG Deilhes F Chaput B Berthier C. Reconstruction of a Dorsal Thoracic Wall Defect With a Dorsal Intercostal Artery Perforator Flap After Removal of a Bulky Cutaneous Squamous Cell Carcinoma: A Case Report. J Med Case Rep (2019) 13(1):294. doi: 10.1186/s13256-019-2226-1 31526388 PMC6747734

[61] Lin C Ballah T Nottage M Hay K Chua B Kenny L. A Prospective Study Investigating the Efficacy and Toxicity of Definitive ChemoRadiation and ImmunOtherapy (CRIO) in Locally and/or Regionally Advanced Unresectable Cutaneous Squamous Cell Carcinoma. Radiat Oncol (2021) 16(1):69. doi: 10.1186/s13014-021-01795-5 33836800 PMC8033693

[62] Migden MR Khushalani NI Chang ALS Lewis KD Schmults CD Hernandez-Aya L. Cemiplimab in Locally Advanced Cutaneous Squamous Cell Carcinoma: Results From an Open-Label, Phase 2, Single-Arm Trial. Lancet Oncol (2020) 21(2):294–305. doi: 10.1016/S1470-2045(19)30728-4 31952975 PMC7771329

[63] Papadopoulos KP Johnson ML Lockhart AC Moore K Falchook GS Formenti SC. First-In-Human Study of Cemiplimab Alone or In Combination With Radiotherapy and/or Low-Dose Cyclophosphamide in Patients With Advanced Malignancies. Clin Cancer Res (2020) 26(5):1025–33. doi: 10.1158/1078-0432.CCR-19-2609 31796520

