# Supplementary material for: A Novel Radiotherapeutic Approach to Treat Bulky Metastases Even From Cutaneous Squamous Cell Carcinoma: Its Rationale and a Look at the Reliability of the Linear-Quadratic Model to Explain Its Radiobiological Effects
Source: Front Oncol. 2022 Feb 23;12:809279. doi: 10.3389/fonc.2022.809279 (PMC8904747; doi:10.3389/fonc.2022.809279)
Supplement: Supplementary file 1 [file DataSheet_1.pdf]

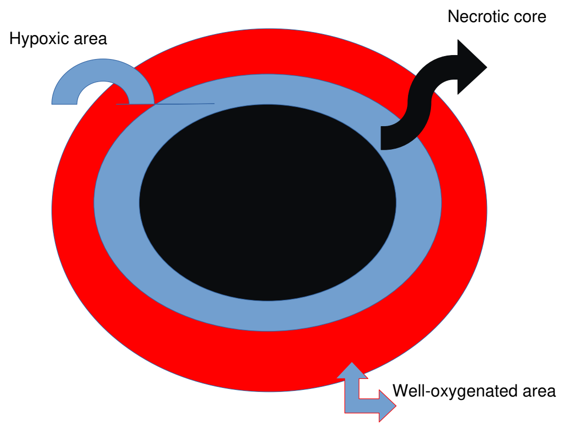

Figure 1: Schematic representation: necrotic core (black), mitotic area (red), hypoxic area (blue)

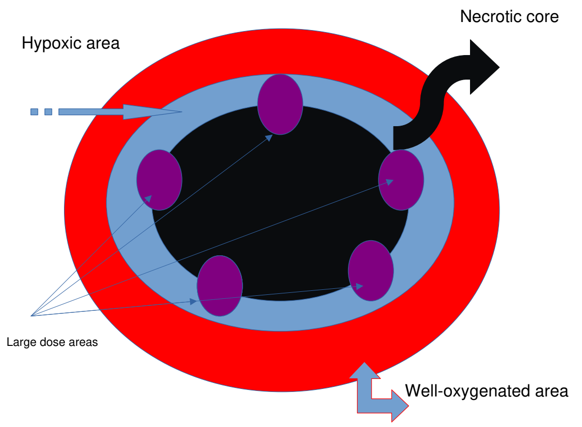

Figure 2: Schematic representation: necrotic core (black), mitotic area (red), whole hypoxic area (blue), vertices with large dose-boost (purple).

Supplementary material: Numerical analysis

I. INTRODUCTION

A quantitative analysis of radiotherapy effects of the specific protocols discussed in the text is carried out. The suggested treatment consists of a large initial dose in the hypoxic subvolumes (vertices), followed by a standard, lower dose, uniformly distributed treatment.

The strategy clarified in the case report section can be summarized and simplified as shown in Figs.(1,2). In Fig.1 the tumor spheroid model of the entire tumor is depicted and in Fig.2 the sub-areas (vertices) of the large boost dose are shown. The vertices are chosen by the radio-oncologist according to the specific patient condition, considering the distance from the OARS and the size of the whole hypoxic area. A conservative approach suggests to choice the vertices in partial overlap with the necrotic core (see Fig. 2), to maintain enough distances from the OARS.

However, due to direct radiation distribution, the effects of the large dose (on the vertices) on the nearby well-oxygenated and hypoxic cells have to be taken into account.

The quantitative analysis is based on the following points:

a) tumor spheroid approximation, since the volumes of the cell sub-populations are more relevant than their shapes;

b) the radiation effects are described by the linear quadratic model;

c) the radiation resitance of the hypoxix area is taken into account by OER approach;

d) vertices are localized in partial overlap with the necrotic area;

e) the effects of the initial large dose (15 Gy) on the normoxic and hypoxic cells is described by average doses;

f) 10 daily doses of 3 Gy follow the initial treatment.

II. NUMERICAL ANALYSIS

A. General setting

For any specific volume with active cells the radiation effects and the regrowth rate can be described by the Gompertz law

$$\frac{1}{V(t)}\frac{dV(t)}{dt} = k_a ln[\frac{V^\infty}{V_a(t)}] - \frac{L}{\tau}, \qquad (1)$$

where k, V^∞ are the usual Gompertz growth parameters, L describes the radiation effect in the linear-quadratic model of a single dose and τ the time interval between doses. Since one considers fast treatments, i.e. with short time interval ($\tau = 1$ day), the effect of the cells regrowth rate can be neglected. Therefore an approximated evaluation of the survival fraction, S ,for constant cell density and after n doses, d in Gy, turns out to be

$$S = V^f/V^i = exp[(-\alpha d - \beta d^2)n] \qquad (2)$$

where (f, i) indicate the final and initial volumes and α and β are the parameters of the linear quadratic model.

Let us recall that the radioresistance depends on the oxygen enhancement ratio (OER) on the dose per fraction, included by assuming a rescaling of hypoxic clonogen subpopulation parameters, $\alpha/\alpha_h = OER$ and $(\alpha/\beta)_h = (\alpha/\beta)OER$, by a factor $OER = 1.5 - 2$.

Therefore, for constant density, by previous equation one gets the survival fraction of the hypoxic cells reported

dose (Gy)	OER=1.5	OER=2.0
3	8.22*10^{-3}	3.17*10^{-2}
5	1.4*10^{-4}	1.93*10^{-3}
7	1.13*10^{-6}	7.9*10^{-5}
10	2.28*10^{-10}	3.05*10^{-7}

Table I: Survival fraction of the hypoxic cells for 10 daily doses for different OER and doses.

in table 1, for $n = 10$, $\alpha = 0.2$ and $\alpha/\beta = 10$ and for different doses d_2 and OER. Table 1 shows that one needs a large dose to obtain a strong tumor control of the hypoxic volume.

For this reason, different methods of radiation delivery have been proposed (see text) and the corresponding survival probability are now approximately evaluated.

According to the tumor spheroid model depicted in fig.1, let us call $V_T^i, V_T^f, V_c^i, V_c^f, V_n^i, V_n^f, V_h^i, V_h^f$ respectively the initial and final volumes of the total system,of the normoxic corona, of the necrotic core and of the hypoxic corona respectively.

The initial volume of the vital cell corona , V_v^i, which contains the normoxic and the hypoxic cells, is given by

$$V_v^i = V_T^i - V_n^i, \tag{3}$$

and therefore

$$V_c^i = V_v^i - V_h^i \tag{4}$$

The initial values here considered are $V_T^i = 171.3$ cm^3, $V_n^i = 86.8$ cm^3, $V_h^i = 13$ cm^3. The radius of the necrotic core turns out to be 2.75 cm, the thickness of the hypoxic corona is $\simeq 0.14$ cm and that one of the normoxic corona is about 0.56 cm.

B. Vertices model

The treatment here discussed is based on the localization of 5 vertices, of single volume 0.4 cm^3, at the boundary of the necrotic area and in partial overlap with the hypoxic and normoxic volumes. The vertices receive a first dose of 15 Gy , followed by the standard treatment of 10 daily doses of 3 Gy, uniformly distributed in the whole tumor volume.

The vertex volume can be written as the sum of the overlap, v_1, with the necrotic core and of the overlapping volumes, v_2 and v_3, with the hypoxic and normoxic spherical coronas.

Moreover, the effects of the initial large dose in the vertices on the other vital cell areas, due to radiation distribution, have to be taken into account. The average dose delivered to the normoxic cell turns out to be $d_{nor} = 4.3$ Gy and $d_{hy} = 5.1$ Gy for hypoxic cells.

According to the previous discussion, the various volumes after the first dose and before the standard treatment are given by:

a) Normoxic volume

$$V_{nor}^{in} = (V_c^i - 5.0v_3)e^{-(\alpha d_{nor}+\beta d_{nor}^2)} + 5.0v_3 e^{-(\alpha * D+\beta * D^2)}, \tag{5}$$

b) Hypoxic volume

$$V_{hy}^{in} = (V_h^1 - 5.0v_2)e^{-(\alpha_h * d_{hy}+\beta_h * d_{hy}^2)} + 5.0v_2 e^{-(\alpha_h * D+\beta_h * D^2)}. \tag{6}$$

By initial values and the geometrical evaluation of v_1, v_2, v_3 one gets $V_{nor}^{in} \simeq 20.7$ cm^3 and $V_{hy}^{in} \simeq 5.05$ cm^3, with $\simeq 70\%$ and $\simeq 60\%$ of reduction of the corresponding initial (no treatment) volumes.

The final volumes after n daily doses, $d = 3$ Gy, are:

$$V_c^f = V_{nor}^{in} e^{-[(\alpha d+\beta d^2)]n}, \tag{7}$$

$$V_h^f = V_{hy}^{in} e^{-[(\alpha_h d+\beta_h d^2)]n}. \tag{8}$$

The survival fractions, obtained by the ratio of the previous volumes for the corresponding initial ones, for $n = 10$, $\alpha = 0.2$, $\beta = \alpha/10$, OER=1.5, are reported in tables 2-3. The final volumes are very small compared to the final tumor volume (113.1 cm^3) and essentially undetectable by metabolic activity also (PET).

C. Hypoxic corona high dose model

In a different proposed treatment (see Tubin et al.) the initial high dose is distributed on the whole hypoxic corona volume (no vertices), producing an average effective dose on the normoxic cell of $d_{nor} = 10.44$ Gy. Therefore, before the standard treatment, the reduced volumes turn out to be

a) Normoxic volume

$$V_{nor}^{in} = V_c^1 e^{-(\alpha d_{nor}+\beta d_{nor}^2)} \simeq 1 \ cm^3 \tag{9}$$

b) Hypoxic volume

$$V_{hy}^{in} = V_h^1 e^{-(\alpha_h D+\beta_h D^2)} \simeq 0.24 \ cm^3 \tag{10}$$

to be compared with the corresponding values $V_{nor}^{in} \simeq$ 20.7 cm^3 and $V_{hy}^{in} \simeq 5.05$ cm^3 of the previous method with 5 vertices.

From this point of view the second method gives a lower survival probability, i.e. a better tumor control probability, at the price of a very large dose ($>$ 10 Gy) distributed in the nearby area.

D. Detailed comparison

A more detailed comparison between the two schemes can be done by evaluating the survival fraction during the

dose	Vertices model	Whole Hypoxic corona model
0	0.29	1.4*10^{-2}
1	0.13	2.94*10^{-3}
2	6.1*10^{-2}	2.94*10^{-3}
3	2.8*10^{-2}	1.35*10^{-3}
4	1.3*10^{-2}	6.2*10^{-4}
5	5.9*10^{-3}	2.8*10^{-4}
6	2.7*10^{-3}	1.3*10^{-4}
7	1.23*10^{-3}	6*10^{-5}
8	5.7*10^{-4}	2.7*10^{-5}
9	2.6*10^{-4}	1.25*10^{-5}
10	1.19*10^{-4}	5.7*10^{-6}

Table II: Survival fraction for normoxic cell evaluated by the two different methods; 0 shows the survival fraction after the initial large dose and before the standard treatment.

dose	Vertices model	Whole Hypoxic corona model
0	0.39	1.83*10^{-2}
1	0.24	1.13*10^{-2}
2	0.15	7*10^{-3}
3	9.2*10^{-2}	4.3*10^{-3}
4	5.7*10^{-2}	2.7*10^{-3}
5	3.5*10^{-2}	1.7*10^{-3}
6	2.2*10^{-2}	1.0*10^{-3}
7	1.35*10^{-2}	6.3*10^{-4}
8	8.3*10^{-3}	3.9*10^{-4}
9	5.16*10^{-3}	2.4*10^{-4}
10	3.2*10^{-3}	1.5*10^{-4}

Table III: Survival fraction for hypoxic cells evaluated by the two different methods; 0 shows the survival fraction after the initial large dose and before the standard treatment.

standard treatment for the normoxic and hypoxic cells. The results are reported in tables 2 and 3. Of course, the initial dose of 15 Gy to the whole hypoxic area (rather than to the vertices) gives a small survival probability, i.e. a better tumor control.
