# Supplementary material for: A Novel Radiotherapeutic Approach to Treat Bulky Metastases Even From Cutaneous Squamous Cell Carcinoma: Its Rationale and a Look at the Reliability of the Linear-Quadratic Model to Explain Its Radiobiological Effects
Source: Front Oncol. 2022 Feb 23;12:809279. doi: 10.3389/fonc.2022.809279 (PMC8904747; doi:10.3389/fonc.2022.809279)
Supplement: Supplementary file 2 [file DataSheet_2.docx]

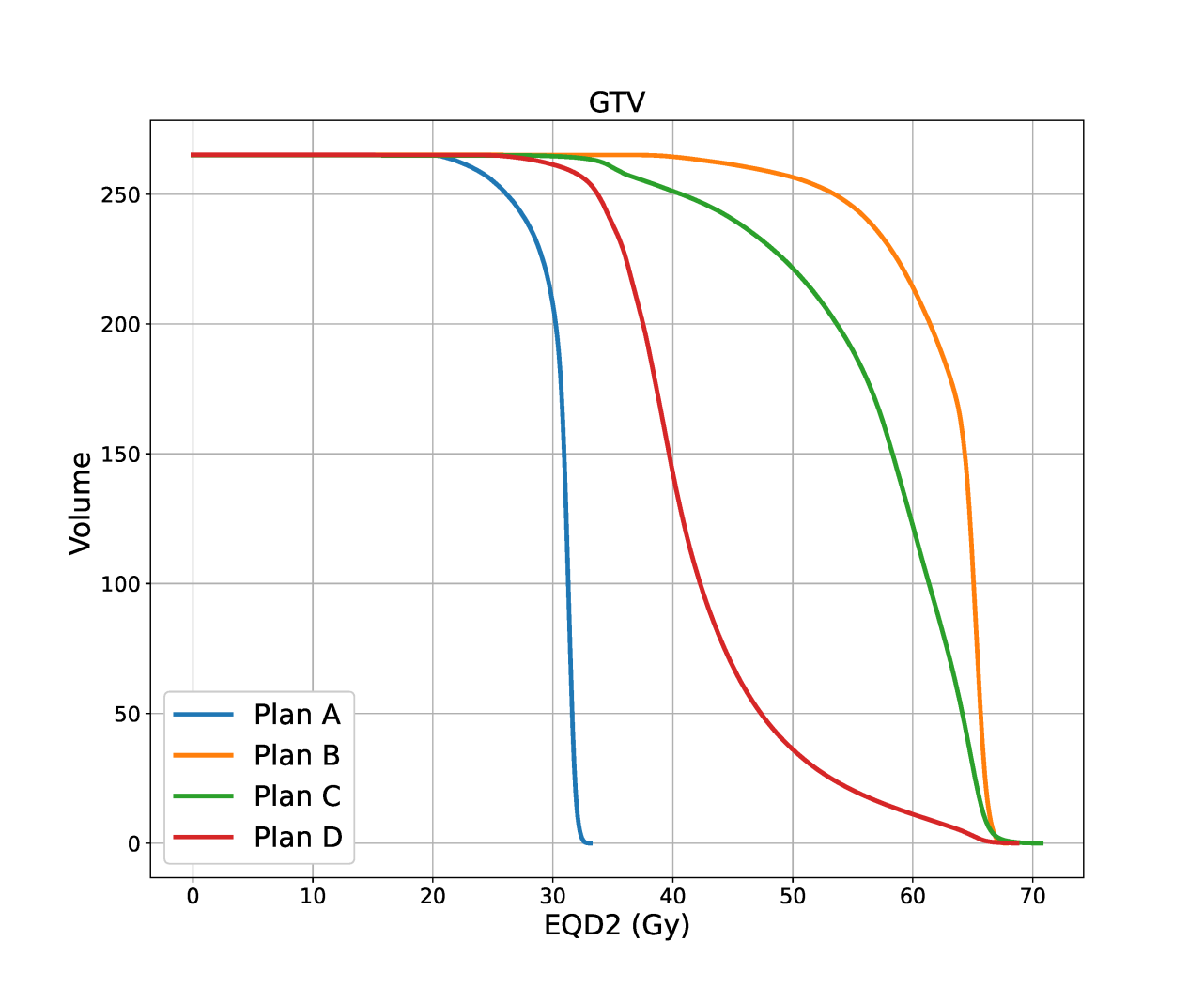


Figura 1 – Cumulative DVH for all plans purposely re-scaled in EQD2: dose-volume distribution for GTV. See the main text for interpretation.


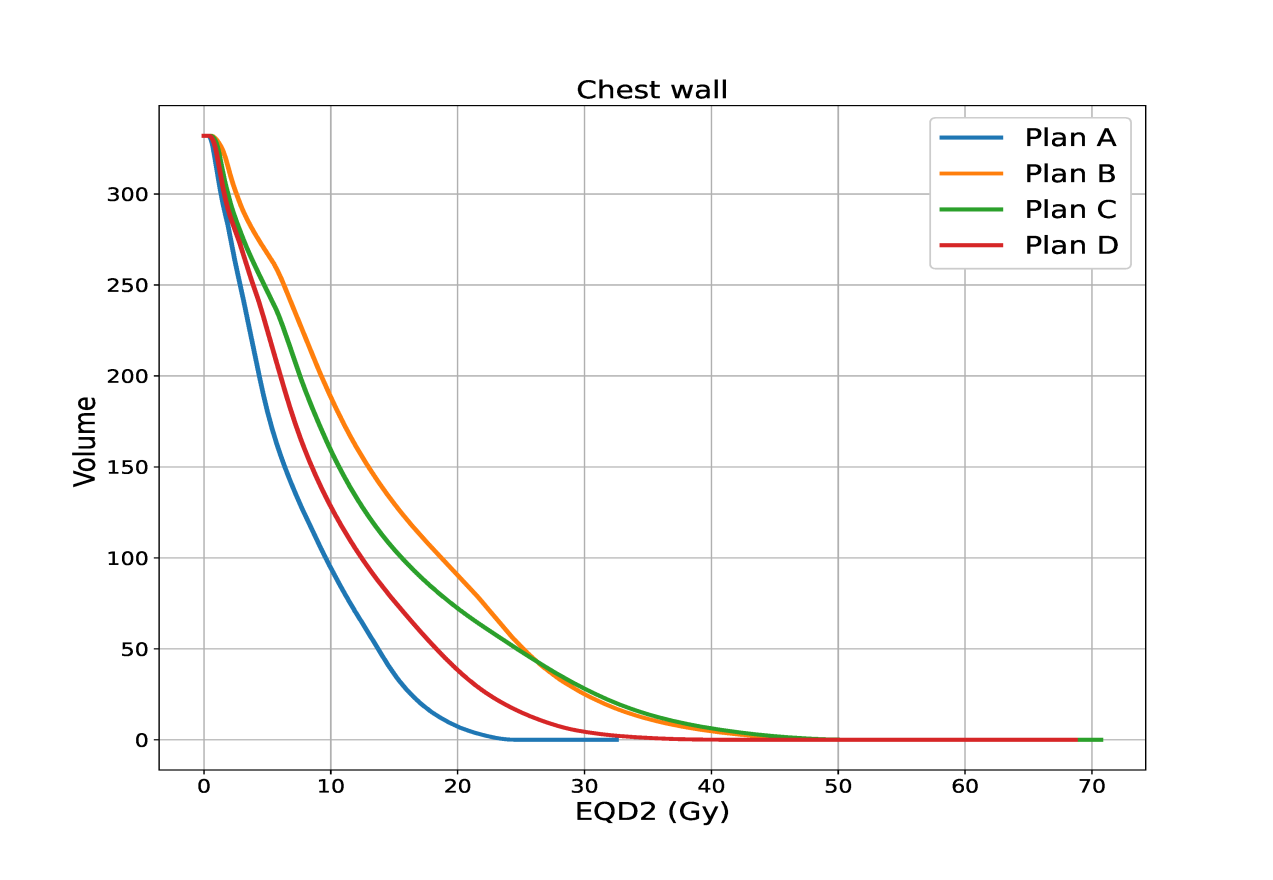


Figura 2 - Cumulative DVH for all plans purposely re-scaled in EQD2: dose-volume distribution for chest wall. See the main text for interpretation.


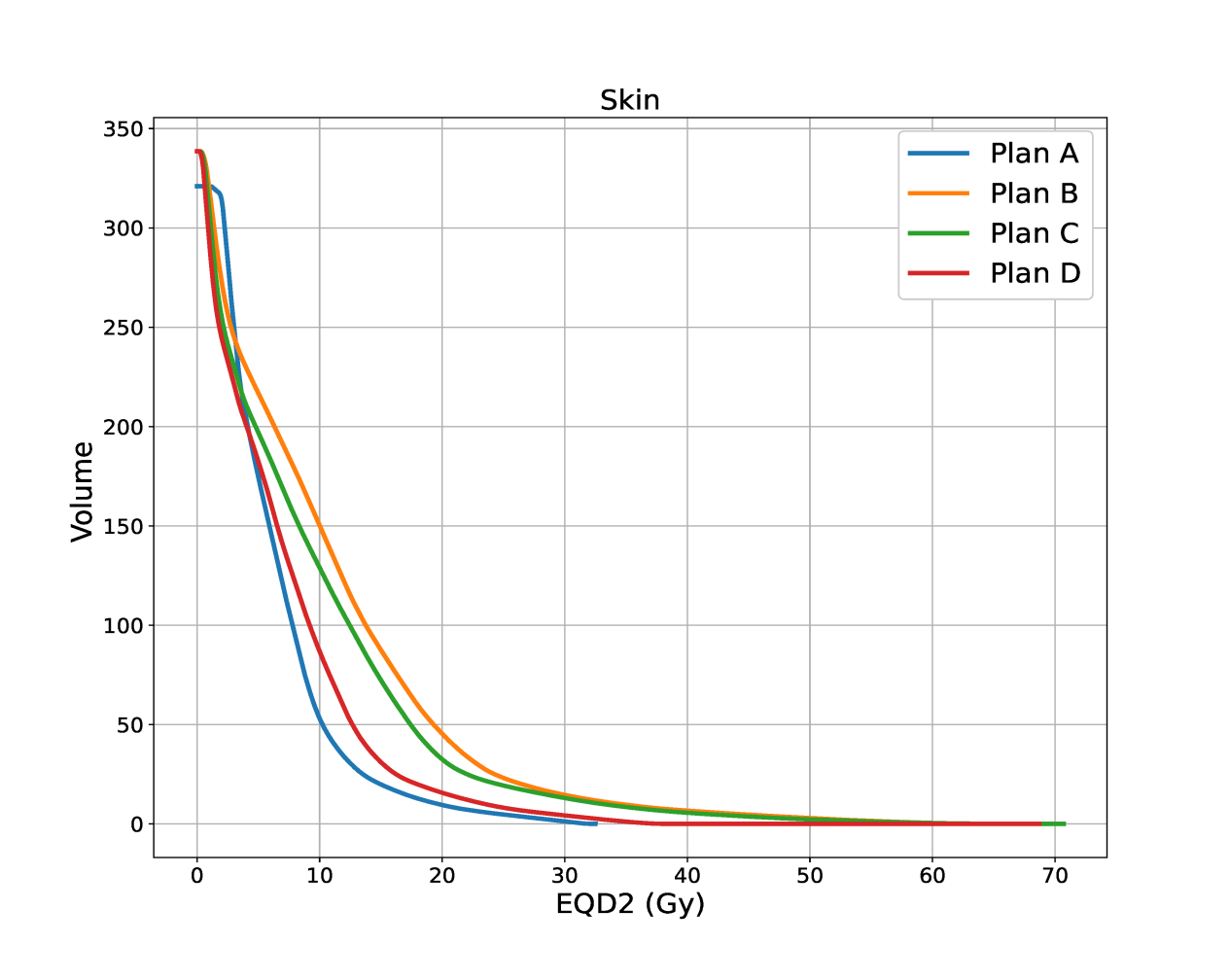


Figura 3 - Cumulative DVH for all plans purposely re-scaled in EQD2: dose-volume distribution for skin. See the main text for interpretation.


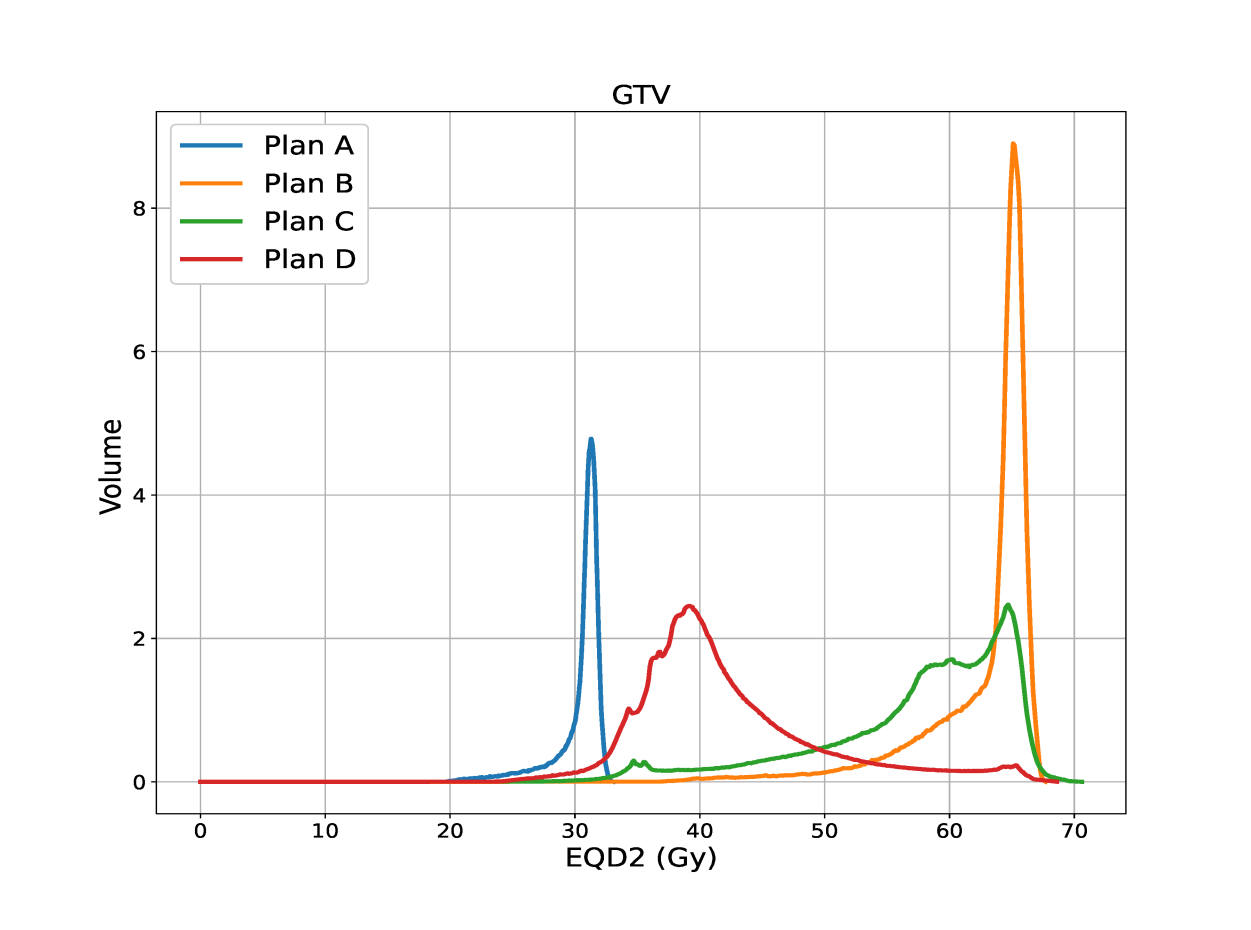


Figura 4 – Differential DVH for all plans purposely re-scaled in EQD2: dose-volume distribution for GTV. See the main text for interpretation.


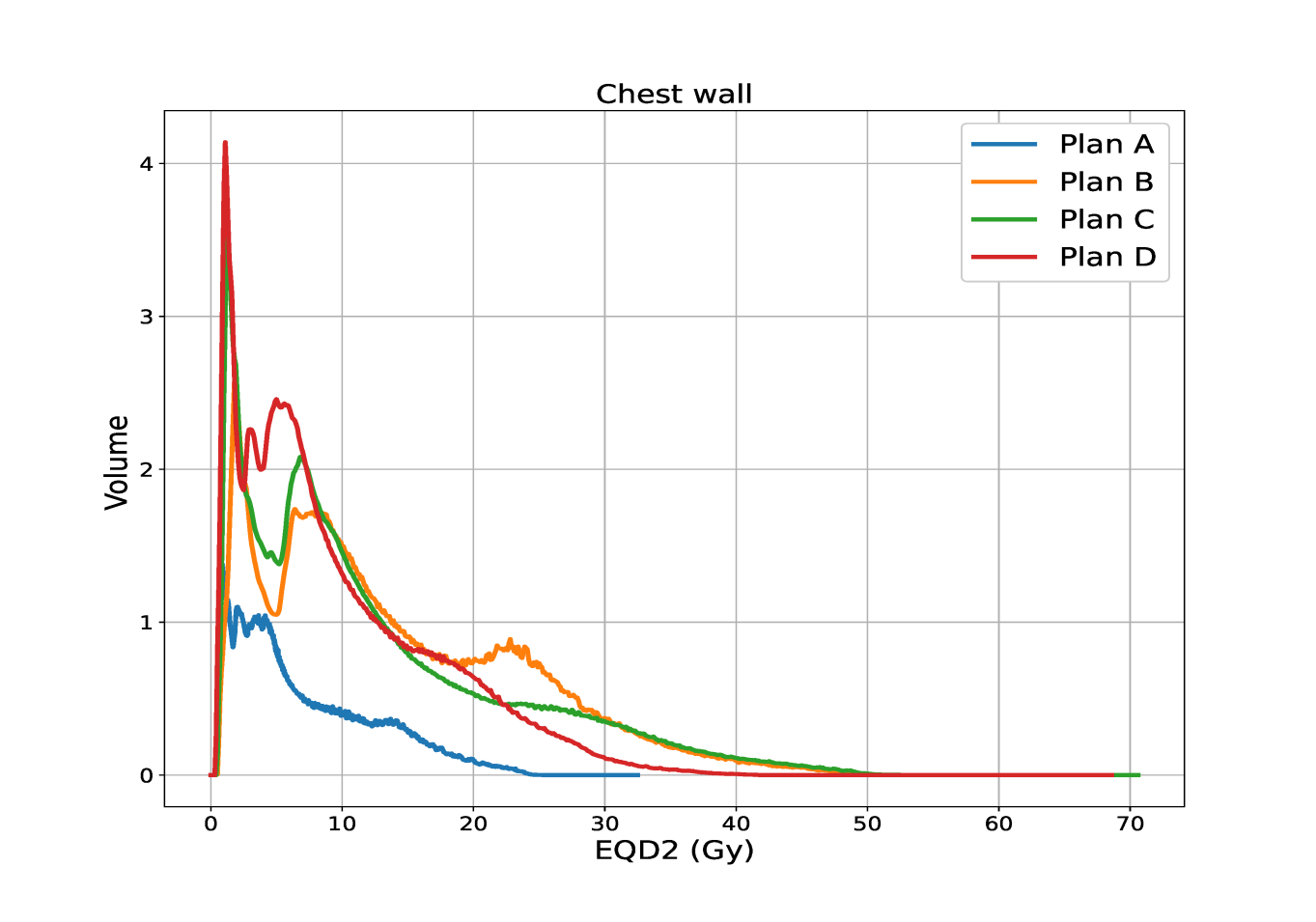


Figura 5 - Differential DVH for all plans purposely re-scaled in EQD2: dose-volume distribution for chest wall. See the main text for interpretation.


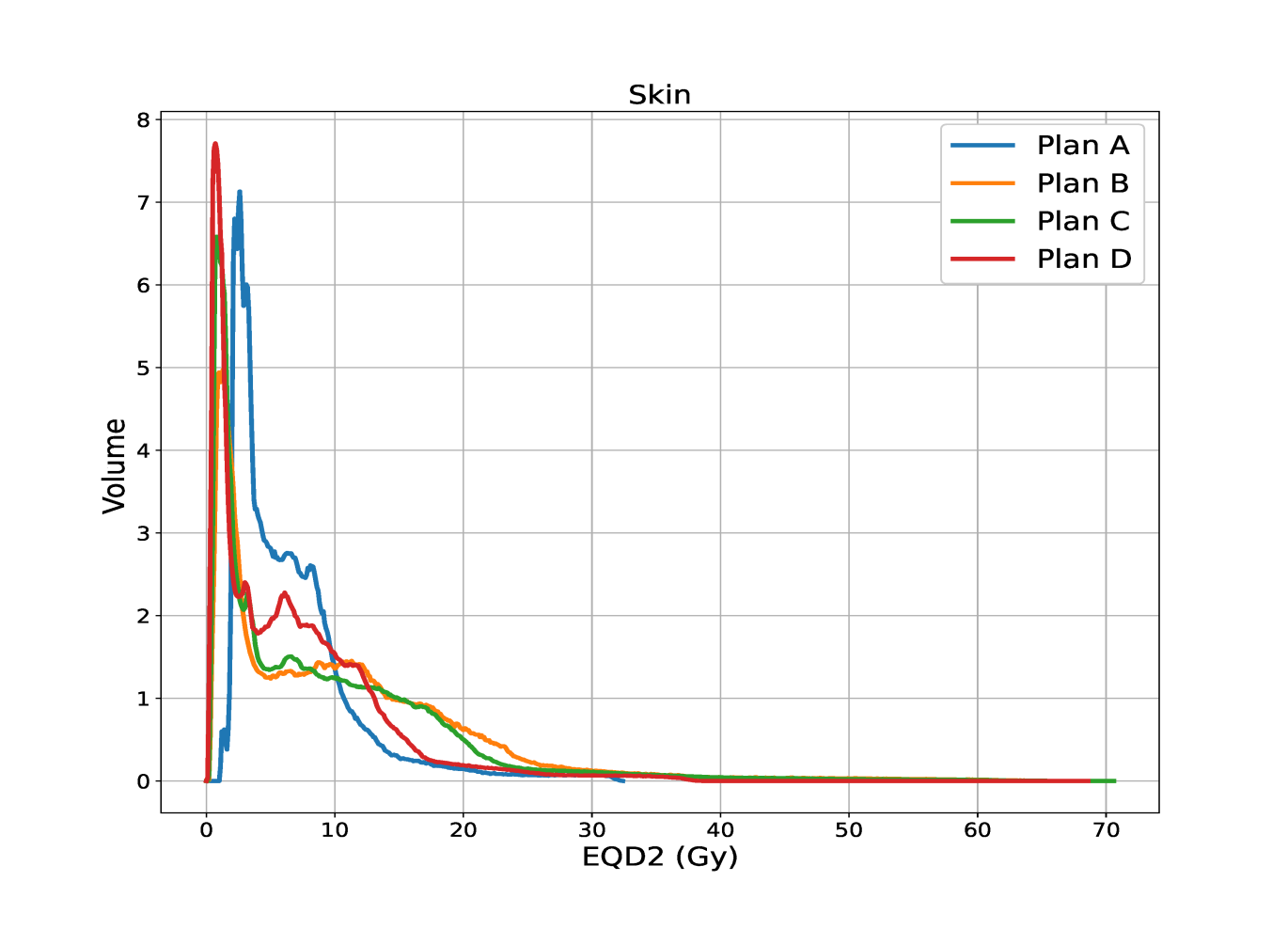


Figura 6 - Differential DVH for all plans purposely re-scaled in EQD2: dose-volume distribution for skin. See the main text for interpretation.
